# Alkylation-based optimization of antifungal FPPS inhibitors yields a potent, broad-spectrum lipophilic zoledronate derivative

**DOI:** 10.1128/mbio.03199-25

**Published:** 2025-12-22

**Authors:** Aidan Kane, Felcia Lai, Joanna G. Rothwell, Thanh Thi Hue Dinh, Leona Campbell, Amy K. Cain, David Hibbs, Jane Hanrahan, Dee Carter

**Affiliations:** 1 School of Life and Environmental Sciences, The University of Sydney 4334, Sydney, NSW, Australia; 2 School of Pharmacy, The University of Sydney 4334, Sydney, NSW, Australia; 3 School of Natural Sciences, ARC Centre of Excellence in Synthetic Biology, Macquarie University, Sydney, NSW, Australia; 4 Sydney Institute for Infectious Diseases, University of Sydney 4334, Sydney, NSW, Australia; Georgia Institute of Technology, Atlanta, Georgia, USA

**Keywords:** bisphosphonate, azole, drug synergy, antifungal therapy, antifungal susceptibility testing, structure-activity relationships, mycology, fungal pathogen

## Abstract

Farnesyl pyrophosphate synthetase (FPPS) is a critical enzyme for squalene biosynthesis. In fungi, squalene is the primary substrate of the ergosterol biosynthesis pathway, which is the target of azole antifungals. Bisphosphonate-based FPPS inhibitors like zoledronate (ZOL) have limited antifungal activity alone but are highly synergistic with azoles against a broad array of human fungal pathogens. Various bisphosphonate drugs are available and are FDA-approved for bone resorption disorders, but their use as antifungal therapeutics is limited by their poor systemic bioavailability. To overcome this, we synthesized a suite of different FPPS inhibitors that incorporated alkyl tails of various lengths to improve solubility, cell penetration, and FPPS inhibition. A derivative of ZOL with an attached 10-carbon alkyl tail (here named 10-ZOL) was also produced. Most of the inhibitors demonstrated limited efficacy and had inconsistent interactions with azole antifungals; however, 10-ZOL had dramatically improved antifungal activity *in vitro* across a broad spectrum of pathogens (minimum inhibitory concentration range = 0.5–8 µg/mL), *in vivo* efficacy in an invertebrate model of infection, and a low level of toxicity in mammalian cells (IC_50_ = 35.77 µg/mL). Rescue with exogenous squalene revealed that 10-ZOL, like ZOL, acts by inhibiting squalene synthesis, resulting in changes to the membrane lipid environment. Cell-based assays showed that these lipid changes compromised plasma membrane structure and integrity and caused an accumulation of toxic oxygen radicals. We conclude that 10-ZOL is a promising new therapeutic lead due to its broad spectrum of activity and synergistic interactions with azoles.

**IMPORTANCE:**

At least 1.6 million lives are lost every year to invasive fungal infections, and topical infections like candidiasis and dermatophytosis are extremely widespread. The current arsenal of antifungal drugs is severely limited; most are either toxic to humans or have a narrow spectrum of activity, and antifungal resistance is an emerging concern. These problems demand new druggable targets with approaches that can mitigate resistance. Here, we build on a body of work that found the disruption of squalene synthesis by bisphosphonates to be a promising approach for synergistically enhancing azole antifungals and preventing the development of resistance. We find an alkylated derivative of the bisphosphonate zoledronate, termed 10-ZOL, to be highly antifungal *in vitro* and *in vivo* against an extremely diverse spectrum of fungal pathogens, with minimal toxicity to mammalian cells.

## INTRODUCTION

Invasive fungal infections are responsible for more than 1.6 million deaths per year, and the number of life-threatening active cases is increasing annually (1). Invasive infections by *Candida, Cryptococcus,* and *Aspergillus* are particularly alarming and are responsible for the majority of fatal mycoses; however, a wide spectrum of fungi can cause serious disease in immunocompromised patients. This diversity complicates and protracts diagnosis and treatment, often resulting in poor patient outcomes (2). Non-fatal mycoses also place a significant burden on public health, and it is estimated that more than one billion people worldwide are currently affected with a dermatophytic infection of the skin, nail, or hair, which can be uncomfortable and cosmetically disfiguring (3).

Unfortunately, the rise in fungal diseases has not been matched by an increase in effective antifungal drugs, and the arsenal of clinically useful antifungals is extremely limited. While there are 18 structural classes of drugs used to treat bacterial infections, there are only five classes of antifungal agents, and these come with severe limitations (4). For example, polyenes such as amphotericin B (AMB) are the only truly broad-spectrum fungicidal agents currently in use, but they can be highly toxic to the kidneys and liver. Azole-based drugs are much better tolerated, but some are fungistatic, and all are ineffective against the Mucorales and other opportunistic molds. Alarmingly, the utility of the few available drugs is being compromised by emerging resistance, largely driven by prolonged prophylactic therapy and the widespread use of fungicides with similar mechanisms of action (5). There is therefore an urgent and unmet need for new broad-spectrum antifungals.

In a previous proteomic study, we found the fungal pathogen *Cryptococcus deuterogattii* upregulates farnesyl pyrophosphate synthetase (FPPS) in response to fluconazole (6), and we have shown that bisphosphonates like zoledronate (ZOL) can inhibit fungal FPPS, resulting in antifungal activity and synergy with azoles against various pathogens (7–10). By inhibiting FPPS, bisphosphonates disrupt the mevalonate pathway and prevent the biosynthesis of squalene, the precursor metabolite in the ergosterol synthesis pathway that is the target of azole antifungals (6). Ergosterol is a vital lipid in the fungal membrane, and a dual assault on the cellular lipid environment by azoles and bisphosphonates critically affects membrane structure and the function of active transporters, which can result in acute oxidative stress (8–10).

Although promising as synergists, bisphosphonates are limited by their propensity to bind to bone mineral (11). This reduces their bioavailability in peripheral tissues, where the burden of invasive infection is highest, restricting their use to superficial or mucosal infections. We therefore sought to improve the pharmacokinetics and bioactivity of ZOL with the addition of a 10-carbon alkyl tail, which has been shown to improve efficacy in a murine malaria model (12–14). We further explored FPPS as an antifungal target by testing a range of alkylated prolyl-aspartic acid derivatives that were predicted to block the substrate-binding channel of human FPPS and had been synthesized and explored as anti-cancer and anti-cholesterol drugs (15). We found the alkylated ZOL derivative, termed 10-ZOL, was by far the most promising FPPS inhibitor, demonstrating broad-spectrum antifungal activity, *in vivo* efficacy, and some synergy with azoles.

## RESULTS

### The antifungal activity of lipophilic FPPS inhibitors and their synergy with azole antifungals

**Fig 1:**
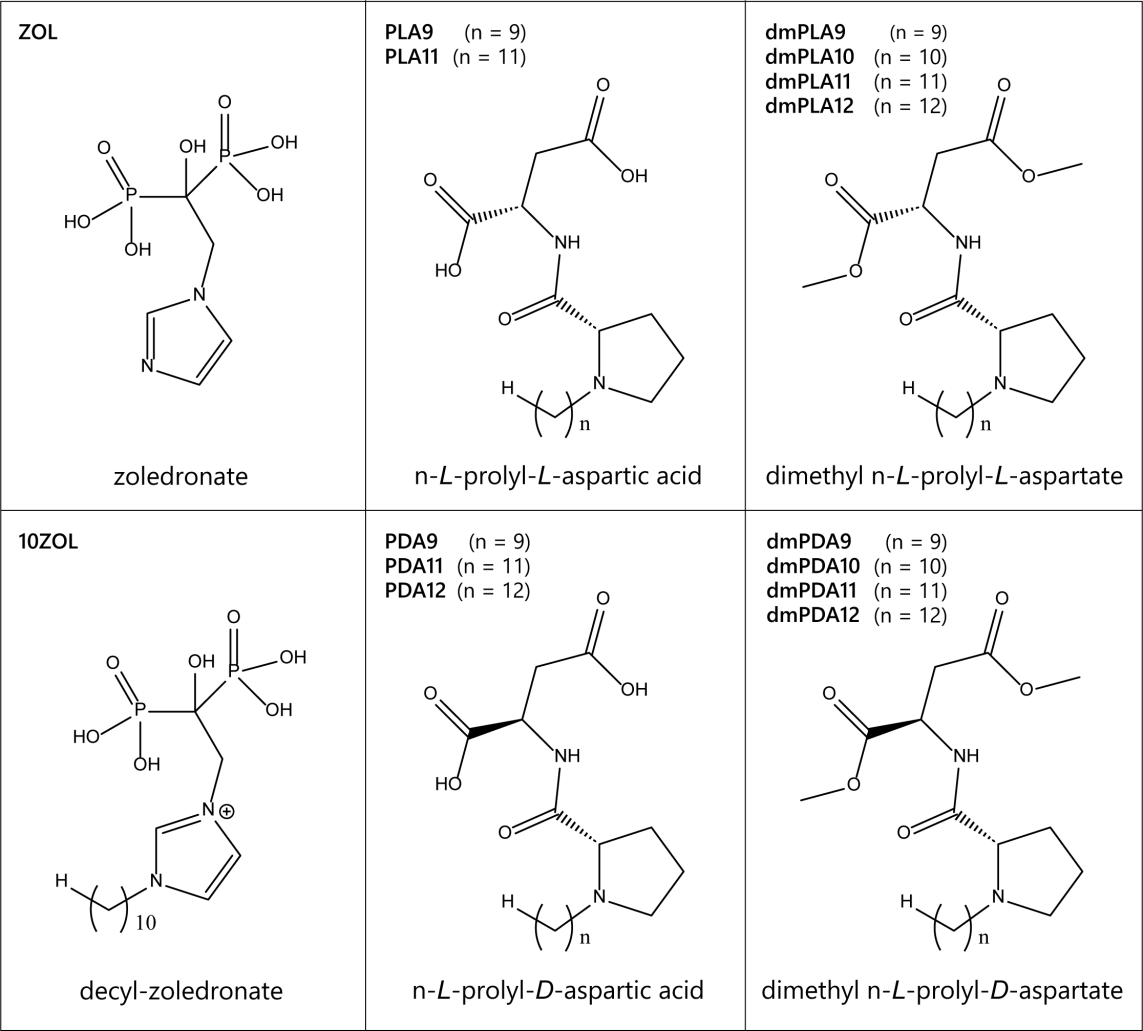
Chemical structures of the lipophilic FPPS inhibitors used in this study. Decyl-zoledronate (10-ZOL) is an analog of ZOL that has been modified to include a 10-carbon tail. All other compounds are derivatives of prolyl-aspartic acid or dimethyl-prolyl-aspartate and have alkyl tails of various lengths.

Small molecules designed to inhibit FPPS included ZOL, a lipophilic derivative of ZOL (10-ZOL), prolyl-aspartic acids, and dimethyl prolyl-aspartic acids (Fig. 1). The antifungal activity of these compounds was evaluated according to CLSI guidelines (16). The minimum inhibitory concentrations (MICs) for each compound and three azole antifungals (fluconazole [FLC], itraconazole [ITR], and ketoconazole [KET]) in eight fungal species are detailed in Table 1. 10-ZOL displayed the most acute inhibitory activity (geometric mean [GM] MIC = 1.2 µg/mL) and was 82-fold more active than unmodified ZOL (GM MIC = 98.7 µg/mL) (*P* < 0.0001), and fungicidal at a 38-fold lower concentration (10-ZOL GM minimum fungicidal concentration [MFC] = 4.8 µg/mL; ZOL GM MFC = 181.0 µg/mL). The range of MICs across the tested fungal species (0.5–4 µg/mL) was also relatively narrow for 10-ZOL compared to ZOL (8–>128 µg/mL). For the prolyl-aspartic acid derivatives, those without methylated carboxyl moieties (PLA9 and 11, and PDA9, 11, and 12) displayed limited or no antifungal activity in any of the species tested, while the dimethyl-prolyl-aspartate derivatives (dmPLA9–12 and dmPDA9–12) were active against some pathogens. Fungicidal activity was observed across all species tested for dmPLA10 (GM MFC = 64.0 µg/mL) and dmPDA10 (GM MFC = 45.3 µg/mL). Other dimethyl-prolyl-aspartate derivatives exhibited antifungal activity in only a narrow range of species.

**TABLE 1:** MIC and MFC values for azoles and lipophilic FPPS inhibitors against a selection of fungal pathogens

Species	Strain	MIC or MFC	MIC/MFC (μg/mL)																	
			FLC	ITR	KET	ZOL	10-ZOL	PLA9	PLA11	dmPLA9	dmPLA10	dmPLA11	dmPLA12	PDA9	PDA11	PDA12	dmPDA9	dmPDA10	dmPDA11	dmPDA12
*Candida albicans*	SC5314	MIC	0.5	0.25	0.0625	128	4	>128	>128	>128	32	32	>128	>128	>128	>128	128	16	>128	>128
		MFC	4	2	0.25	>128	16	>128	>128	>128	32	32	>128	>128	>128	>128	>128	32	>128	>128
*Candida glabrata*	CBS138	MIC	4	0.125	0.5	8	0.5	>128	>128	128	32	32	>128	>128	>128	>128	128	32	128	128
		MFC	>128	4	4	32	1	>128	>128	>128	32	64	>128	>128	>128	>128	>128	32	128	128
*Cryptococcus neoformans*	H99	MIC	2	0.5	0.25	32	1	>128	>128	128	16	16	16	>128	>128	>128	32	16	16	8
		MFC	>128	4	4	>128	2	>128	>128	>128	64	64	16	>128	>128	>128	128	32	32	32
*Cryptococcus deuterogattii*	R265	MIC	2	1	4	128	1	>128	>128	128	16	8	8	>128	>128	>128	64	16	16	8
		MFC	>128	4	8	128	2	>128	>128	>128	128	128	8	>128	>128	>128	64	64	128	32
*Saccharomyces cerevisiae*	S288c	MIC	4	0.25	1	>128	1	>128	>128	32	8	8	4	>128	>128	>128	64	16	64	64
		MFC	64	1	8	>128	8	>128	>128	32	16	8	4	>128	>128	>128	128	16	128	>128
*Aspergillus fumigatus*	ATCC204305	MIC	>128	0.125	4	>128	1	>128	>128	128	32	128	32	>128	>128	>128	128	32	>128	128
		MFC	>128	4	16	>128	2	>128	>128	>128	128	>128	>128	>128	>128	>128	>128	32	128	>128
*Aspergillus flavus*	ATCC204304	MIC	64	0.5	2	>128	2	>128	>128	>128	64	>128	>128	>128	>128	>128	>128	64	>128	>128
		MFC	>128	2	8	>128	4	>128	>128	>128	64	>128	>128	>128	>128	>128	>128	64	>128	>128
*Trichophyton rubrum*	213	MIC	64	8	4	128	1	>128	>128	128	8	16	16	>128	>128	128	32	8	16	8
		MFC	>128	>16	>16	>128	64	>128	>128	>128	>128	>128	>128	>128	>128	>128	>128	>128	>128	>128
Geometric mean		MIC	8.7	0.5	1.3	98.7	1.2	>128	>128	128.0	20.7	29.3	38.1	>128	>128	234.8	83.0	20.7	69.8	49.4
		MFC	128.0	3.7	6.7	181.0	4.8	>128	>128	197.4	64.0	83.0	69.8	>128	>128	>128	181.0	45.3	139.6	139.6

**Fig 2:**
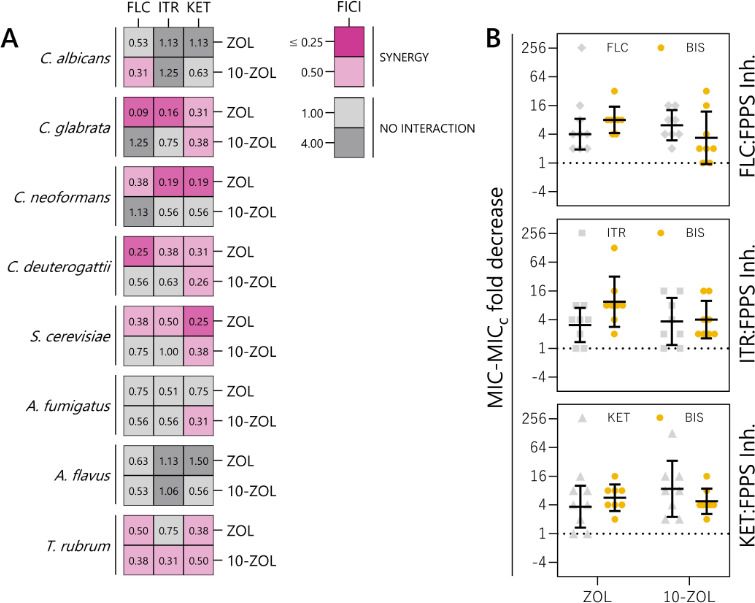
10-ZOL has fewer synergistic interactions with azoles compared to unmodified ZOL; however, combining azoles and bisphosphonates lowers the effective concentrations of both drugs. (**A**) Heatmaps displaying the FICI values for ZOL and 10-ZOL when combined with azole antifungals FLC, ITR, and KET against various fungal pathogens. FICI values are the means of three biological replicates. (**B**) Fold decrease in MIC for each azole and ZOL or 10-ZOL when used in combination compared to when used alone. Each point represents the fold decrease for a representative strain of each fungal species tested; positive values >1 indicate a reduction in effective dosage. MICs, MIC_c_s, and fold decreases are detailed in Table S1.

Synergy between lipophilic FPPS inhibitors and azole antifungals was investigated by determining the fractional inhibitory concentration index (FICI) (Fig. 2; Table S1 Fig. S1) (17). Of the FPPS inhibitors, ZOL exhibited synergy at the lowest concentrations relative to its MIC when combined with FLC, ITR, and KET. Synergy (FICI ≤ 0.25) between relatively low concentrations of ZOL and the azoles was observed in *C. glabrata, C. neoformans, C. deuterogattii,* and *S. cerevisiae* (Fig. 2A). 10-ZOL, while significantly more antifungal than ZOL, did not interact synergistically with most azoles, but it did synergize with KET in *C. glabrata, C. deuterogattii, S. cerevisiae,* and *A. fumigatus*. 10-ZOL was synergistic with all three azoles in *T. rubrum*, while ZOL was synergistic with FLC and KET only. Some interactions between azoles and non-bisphosphonate FPPS inhibitors were antagonistic (FICI ≥ 4), and ZOL, 10-ZOL, and dmPLA11 were the only FPPS inhibitors tested that had no antagonism with any of the azole drugs in any species tested (Fig. S1A). Derivatives PLA9 and 11, and PDA9, 11, and 12 antagonized azoles in *C. albicans, C. neoformans,* and *C. deuterogattii*, yet were largely synergistic in *C. glabrata* and *S. cerevisiae*. At >256, the MIC for FLC in *A. fumigatus* is above what is considered clinically viable, and while not synergistic, adding 10-ZOL reduced the effective concentration of FLC 16-fold to 8 µg/mL (Table S1). ZOL and 10-ZOL were the only FPPS inhibitors that consistently reduced the MIC of the azole when used in combination across all species tested, even in the absence of synergy (Fig. 2B, Fig. S1B).

### Cytotoxicity of lipophilic FPPS inhibitors in mammalian cells

**Fig 3:**
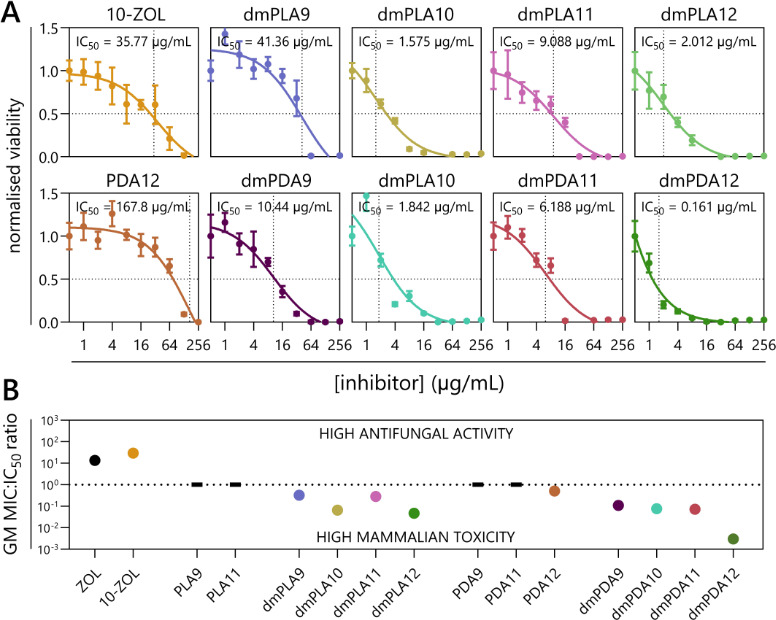
ZOL and 10-ZOL are less toxic to human epithelial cells than they are to fungal pathogens, but the reverse is seen for the lipophilic prolyl-aspartic acid derivatives. (**A**) Metabolic dose-response curves for HEK-293T cells treated with the indicated inhibitors. The IC_50_ represents the concentration at which the metabolic activity of HEK-293T cells was reduced by half, compared to no-drug control (1% DMSO) cells. Data represent the means of six replicates ± SD. Any inhibitors not shown were non-toxic to HEK-293T cells at 256 µg/mL. (**B**) Ratios between the GM of fungal MICs (Table 1) and HEK-293T IC_50_s. Compounds with MICs or IC_50_s above the concentrations tested are represented with a black dash.

The cytotoxicity of each lipophilic FPPS inhibitor in HEK-293T cells was determined with a 3-(4,5-dimethyl thiazol-2-yl)-2,5-diphenyltetrazolium bromide (MTT) reduction assay. Dose-response curves were used to calculate IC_50_, as detailed in Fig. 3A. PLA9, PLA11, PDA9, PDA11, and ZOL (Fig. S2) were non-inhibitory at the maximum concentration tested (256 µg/mL). 10-ZOL (IC_50_ = 35.77 µg/mL) was at least 37.0-fold more cytotoxic than the projected value for ZOL (predicted IC_50_ = 1323 µg/mL) (Fig. S2). Figure 3B shows the ratios between antifungal activity (GM MIC across all eight species tested) and mammalian cell toxicity (IC_50_ in HEK-293T cells) (Table S2). ZOL and 10-ZOL were the only FPPS inhibitors with positive ratios, indicating that they were more inhibitory to fungi than they were to human cells.

### Antifungal activity of 10-ZOL in biofilms formed by pathogenic fungi

To further investigate the antifungal activity of 10-ZOL, fungal biofilms were exposed to 10-ZOL alone and in combination with the azole antifungals. The resulting sessile IC_80_ (SIC_80_), combination sessile IC_80_ (SIC_c_), and sessile FICI (SFICI) values are presented in Table 2. 10-ZOL, when used alone, exhibited inhibitory activity against all biofilms tested (SIC_80_ range 8–128 µg/mL) and was particularly active against biofilms of *C. glabrata* (SIC_80_ = 8 µg/mL). 10-ZOL also reduced the dose of azole drugs required to inhibit biofilms when used in combination by 2–>4-fold. In combination, 10-ZOL potentiated the antifungal activity of FLC against biofilms of *C. glabrata* and *A. fumigatus,* and of ITR for biofilms of *T. rubrum*, where these azoles, when used alone, were non-inhibitory at the concentrations tested. Combining 10-ZOL with azoles resulted in at least a twofold decrease in SIC_80_ for both agents in all fungal biofilms, and for most combinations, the SIC_c_ of 10-ZOL was below its toxic IC_50_ in mammalian HEK-293T cells. Despite this global reduction in inhibitory dosages, however, actual synergy between azoles and 10-ZOL was only consistently observed for biofilms of *Trichophyton rubrum* (FICI range 0.38–0.50).

**TABLE 2:** SIC_80_ and SFICI values for 10-ZOL alone and in combination with azole antifungals

Species	Strain	SIC80 (µg/mL)				FLC:10-ZOL			ITR:10-ZOL			KET:10-ZOL		
						SICc (µg/mL)		SFICI^*a*^	SICc (µg/mL)		SFICI^*a*^	SICc (µg/mL)		SFICI^*a*^
		FLC	ITR	KET	10-ZOL	FLC	10-ZOL		ITR	10-ZOL		KET	10-ZOL	
*Candida albicans*	SC5314	512	256	64	128	128	32	*0.50*	128	64	1.00	32	64	1.00
*Candida glabrata*	CBS138	>2,048	256	256	8	512	4	0.63	128	4	1.00	64	2	*0.50*
*Cryptococcus neoformans*	H99	256	64	32	128	128	32	0.75	32	16	0.63	16	8	0.56
*Cryptococcus deuterogattii*	R265	256	128	256	64	128	32	1.00	64	32	1.00	32	16	*0.38*
*Saccharomyces cerevisiae*	S288c	256	32	32	64	64	32	0.75	16	32	1.00	16	8	0.63
*Aspergillus fumigatus*	ATCC 204305	>2,048	256	256	128	512	64	0.63	128	32	0.75	64	16	*0.38*
*Aspergillus flavus*	ATCC 204304	1,024	128	128	128	512	16	0.63	64	64	1.00	64	32	0.75
*Trichophyton rubrum*	213	2,048	>256	256	64	256	16	*0.38*	64	16	*0.38*	64	16	*0.50*

^*a*^ Values that indicate a synergistic drug interaction have been italicized.

### 10-ZOL inhibits squalene synthesis, causing a depletion of ergosterol and a fungicidal effect

**Fig 4:**
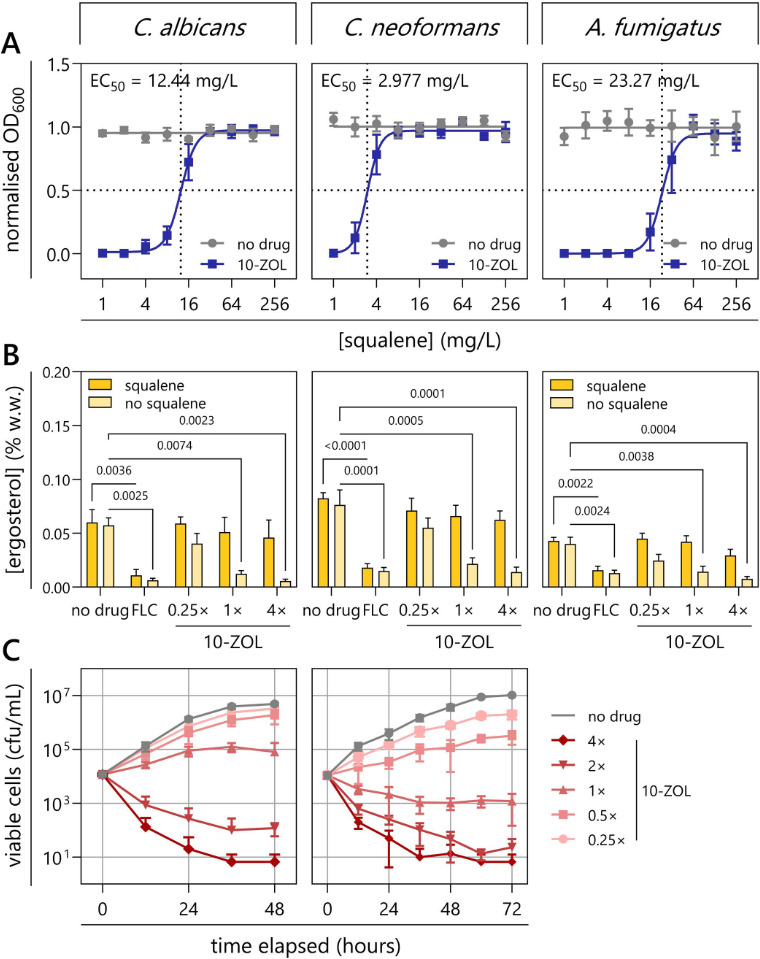
10-ZOL inhibits squalene synthesis, causing the depletion of ergosterol and a fungicidal effect. (**A**) Squalene rescue assays. *C. albicans* SC5314, *C. neoformans* H99, and *A. fumigatus* ATCC 204305 were treated with 10-ZOL at the MIC or with a no-drug control (1% DMSO). Exogenous squalene was added at the indicated concentrations. EC_50_ is the concentration of squalene that rescued 50% of fungal growth relative to the no-drug control. Data are the mean of three replicates ± SD. (**B**) Cell ergosterol content following treatment with FLC or with 10-ZOL at 0.25, 1, and 4× MIC, with and without exogenous squalene (128 µg/mL). Ergosterol concentration was calculated as a percentage of the total wet weight of treated fungi. Data are the mean of three technical replicates of each of three independent biological replicates ± SD. Treatments with or without squalene were compared by two-way ANOVA, and significant (<0.05) *P*-values are presented. (**C**) Time-kill assays for *C. albicans* SC5314 and *C. neoformans* H99 treated with a no-drug control (1% DMSO) or 10-ZOL at 0.25, 0.5, 1, 2, or 4× MIC. Data are the mean of four technical replicates of each of three independent biological replicates ± SD.

The role of squalene in the antifungal effect of 10-ZOL was determined using a squalene rescue assay in representative strains of three common invasive fungal pathogens: *Candida albicans*, *Cryptococcus neoformans*, and *Aspergillus fumigatus* (Fig. 4A). Exogenous squalene successfully restored growth of all three fungal pathogens in a dose-dependent manner, indicating that squalene synthesis plays an essential role in the antifungal effect of 10-ZOL. The rescue EC_50_ had no correlation with the 10-ZOL MIC across the three organisms (*r* = −0.039, *P* = 0.975).

Quantification of cellular ergosterol found that 10-ZOL treatment depleted ergosterol in the fungal membrane (Fig. 4B). FLC treatment alone caused a substantial reduction in membrane ergosterol that could not be rescued by the addition of squalene, consistent with FLC inhibition occurring downstream from squalene synthesis. Compared to the no-drug control (1% DMSO), 10-ZOL caused significant depletion of membrane ergosterol at 1× MIC in *C. albicans* (*P* = 0.0084), *C. neoformans* (*P* = 0.0005), and *A. fumigatus* (*P* = 0.0038). No significant difference in ergosterol content was observed in cells treated with FLC and cells treated with 10-ZOL at 1 or 4× MIC. Supplementation with squalene caused a significant rescue of ergosterol content in cells treated with 10-ZOL at 1× MIC in *C. albicans* (*P* = 0.0325) and *C. neoformans* (*P* = 0.0057), and at 0.25× MIC in *A. fumigatus* (*P* = 0.0328). There was no significant difference in ergosterol content observed between untreated cells and cells that had been treated with 10-ZOL at any concentration and rescued with squalene. Overall, these results confirmed that the disruption of squalene synthesis and subsequent depletion of ergosterol in the fungal membrane were vital components of the antifungal mechanism of 10-ZOL.

A time-kill assay for 10-ZOL further demonstrated its fungicidal properties in yeast pathogens *C. albicans* and *C. neoformans* (Fig. 4C). Following treatment at 1× MIC, *C. albicans* cultures grew slowly until 24 h post-treatment and viability began to decline at 48 h, while at 2× MIC the viable cell count had decreased significantly from the starting inoculum at 12 h of treatment (*P* = 0.0207). For *C. neoformans,* 10-ZOL at 1× MIC decreased viable cell count immediately post-treatment, becoming significantly different to the starting inoculum at 24 h (*P* = 0.0120).

### 10-ZOL-mediated ergosterol depletion results in reduced fungal membrane structure and integrity

**Fig 5:**
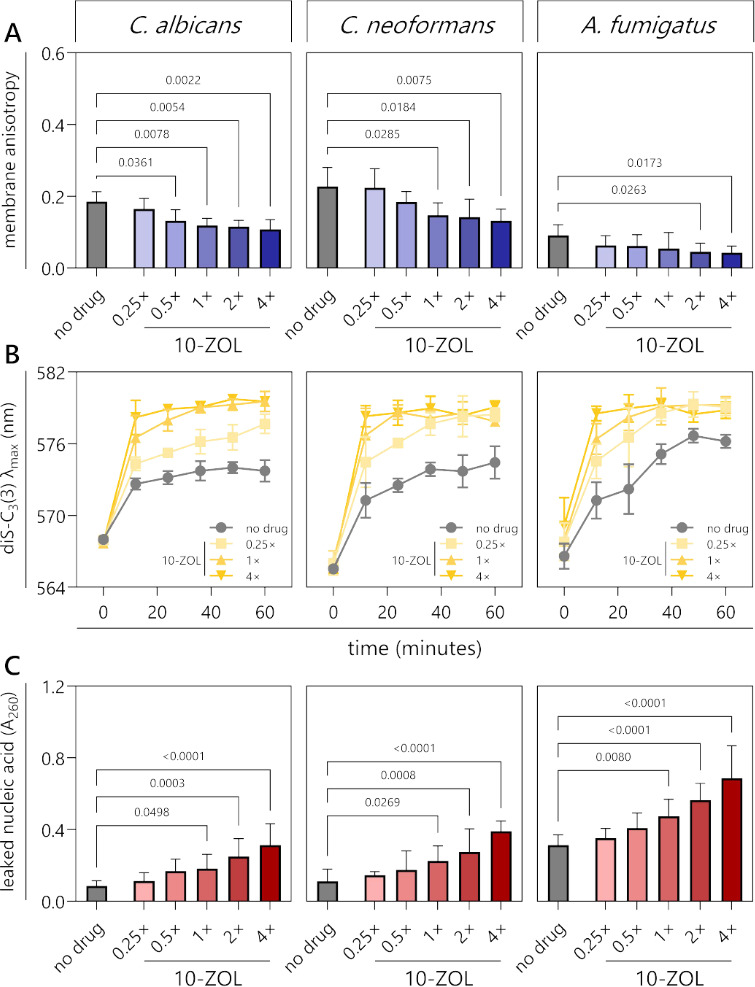
10-ZOL treatment causes hyperfluidity, depolarization, and leakage in fungal membranes. (**A**) Membrane hyperfluidity is expressed as the fluorescence anisotropy of TMA-DPH. *C. albicans* SC5314, *C. neoformans* H99, and *A. fumigatus* ATCC 204305 were treated with a no-drug control (1% DMSO) or 10-ZOL at 0.25, 0.5, 1, 2, or × MIC. (**B**) Fungal membrane depolarization is expressed as the changing emission λ_max_ of diS-C_3_(3) over time. (**C**) Membrane permeabilization is measured by the amount of nucleic acid leaked into the culture supernatant. Data are the mean of three technical replicates of each of three biological replicates ± SD. Treatments were compared to the no-drug control (1% DMSO) by one-way ANOVA, and significant (<0.05) *P*-values are presented.

The fluorescence anisotropy of 1-(4-trimethylammoniumphenyl)-6-phenyl-1,3,5-hexatriene p-toluenesulfonate (TMA-DPH), a membrane-associated probe, was measured to investigate the effect of 10-ZOL on fungal membrane fluidity. Greater anisotropy indicates a more ordered membrane structure. 10-ZOL caused a dose-dependent decrease in anisotropy (and therefore an increase in fluidity) compared to the no-drug control in *C. albicans* (*r* = −0.7561, *P* = 0.0420), *C. neoformans* (*r* = −0.8128, *P* = 0.0293), and *A. fumigatus* (*r* = −0.7662, *P* = 0.0456). Treated membranes were significantly different from the no-drug control membranes starting at 0.5× MIC in *C. albicans* (*P* = 0.0361), 1× MIC in *C. neoformans* (*P* = 0.0285), and 2× MIC in *A. fumigatus* (*P* = 0.0263) (Fig. 5A).

Membrane depolarization in 10-ZOL-treated fungi was measured using diS-C_3_(3), a probe that accumulates in depolarized membranes, causing a red shift in its emission wavelength and an increase in fluorescence (18). In all three species, 10-ZOL treatment affected membrane polarity, causing a rapid increase in the diS-C_3_(3) emission maximum, indicating a dose-dependent depolarization effect (Fig. 5B).

The effect of 10-ZOL on membrane permeability was further evaluated by quantifying the leakage of nucleic acid into the supernatant (Fig. 5C) (19). 10-ZOL had a dose-dependent effect on permeabilization in *C. albicans* (*r* = 0.9554, *P* = 0.0029), *C. neoformans* (*r* = 0.9830, *P* = 0.0004), and *A. fumigatus* (*r* = 0.9773, *P* = 0.0008). A significant increase in leaked nucleic acid was observed in cells treated with 1× MIC or higher in *C. albicans* (*P* = 0.0498), *C. neoformans* (*P* = 0.0270), and *A. fumigatus* (*P* = 0.0080).

### 10-ZOL causes the accumulation of ROS and induces acute oxidative stress in fungal pathogens

**Fig 6:**
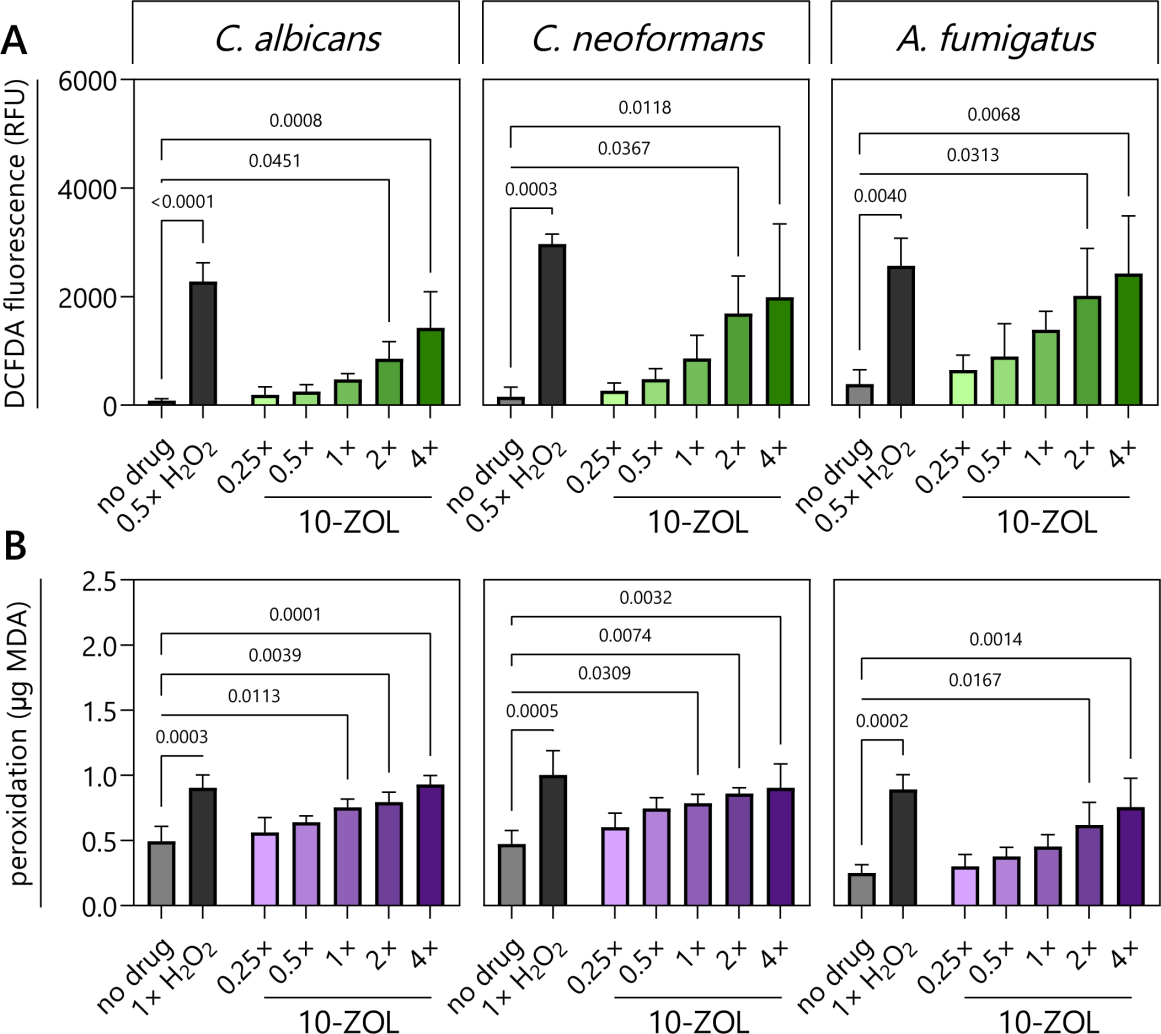
10-ZOL causes the accumulation of intracellular ROS and results in oxidative damage to fungal cells. (**A**) Intracellular ROS accumulation as indicated by the fluorescence of DCFDA. H_2_O_2_ at 0.5× MIC was included as a positive control. (**B**) Lipid peroxidation in 10-ZOL-treated fungi, assessed by the appearance of malondialdehyde (MDA) and normalized to the total protein content of cell lysates. Data are the mean of three technical replicates of each of three biological replicates. Treatments were compared to the no-drug control (1% DMSO) by one-way ANOVA, and significant (<0.05) *P*-values are presented.

The accumulation of intracellular reactive oxygen species (ROS) was measured using 2′,7′-dichlorodihydrofluorescein diacetate (DCFDA), a fluorescent ROS indicator (Fig. 6A). H_2_O_2_, which causes the build-up of toxic oxygen radicals, was used as a positive control. 10-ZOL had a dose-dependent effect on ROS accumulation in *C. albicans* (*r* = 0.9907, *P* = 0.0002), *C. neoformans* (*r* = 0.9488, *P* = 0.0039)*,* and *A. fumigatus* (*r* = 0.9479, *P* = 0.0040) (Fig. 6A). The increase in fluorescence became significant at 2× MIC for *C. albicans* (*P* = 0.0451), *C. neoformans* (*P* = 0.0367), and *A. fumigatus* (*P* = 0.0313).

The extent of oxidative damage caused by 10-ZOL treatment was assessed by measuring lipid peroxidation (Fig. 6B). 10-ZOL caused dose-dependent oxidative damage in *C. albicans* (*r* = 0.9343, *P* = 0.0063), *C. neoformans* (*r* = 0.8151, *P* = 0.0481)*,* and *A. fumigatus* (*r* = 0.9704, *P* = 0.0013). The increase became significant at 1× MIC in *C. albicans* (*P* = 0.0113) and *C. neoformans* (*P* = 0.0309) and at 2× MIC in *A. fumigatus* (*P* = 0.0167).

### 10-ZOL is antifungal against a broad spectrum of clinically relevant fungal species

**Fig 7:**
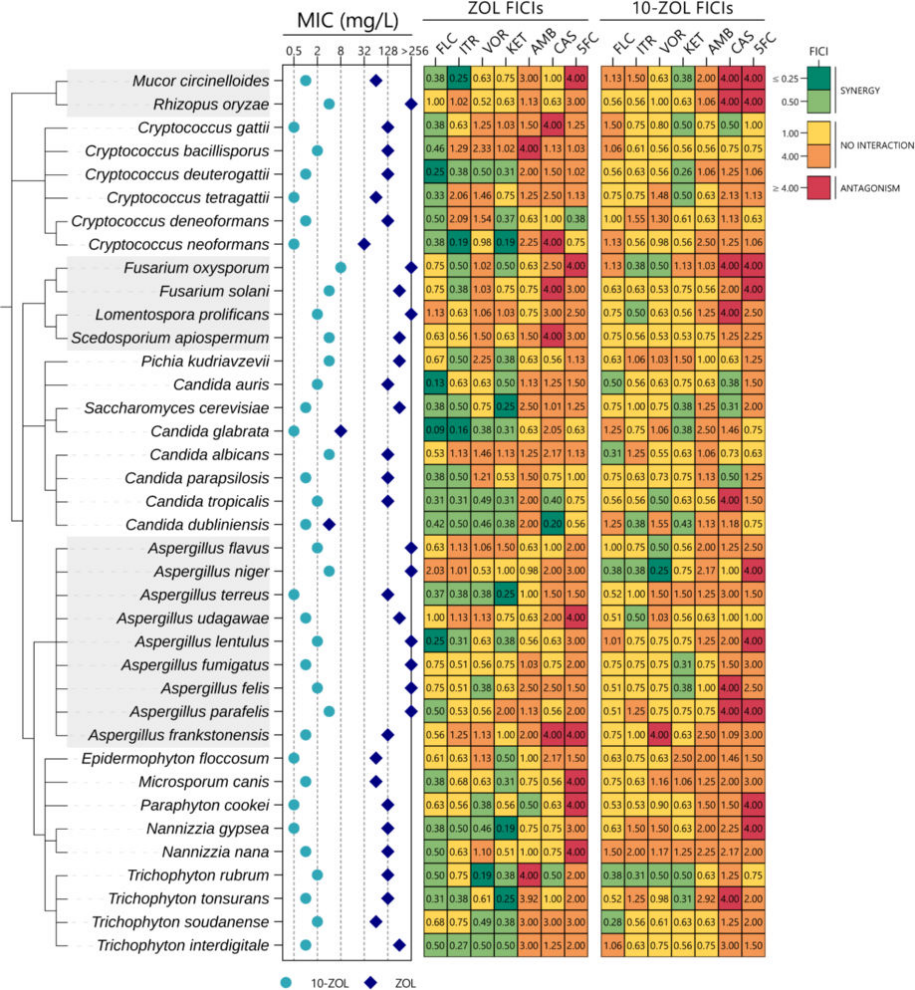
10-ZOL is more active than ZOL against a broad spectrum of fungal species, but less synergistic with antifungals. The MIC for both ZOL and 10-ZOL against one strain from each of 38 clinically relevant fungal species is presented, alongside heatmaps displaying the FICIs for ZOL and 10-ZOL when combined with seven commonly used antifungals. Note that the true FICIs for antagonistic pairings may be higher than those presented, as 4.00 was the maximum FICI obtainable with the screening methods used.

To assess the spectrum of activity of 10-ZOL, MICs were obtained for 38 species of clinically relevant fungi (Fig. 7; Table S3). For each fungal species tested, the 10-ZOL MIC was lower than the ZOL MIC, with an overall MIC range of 0.5–8 µg/mL for 10-ZOL compared to 4–>256 µg/mL for ZOL, and a GM MIC for 10-ZOL that was 106.7-fold lower than that of ZOL (1.39 ± 1.6 µg/mL vs*.* 148.11 ± 176.3 µg/mL). When combined with azoles, ZOL was consistently synergistic at lower relative concentrations than 10-ZOL (Fig. 7; Fig. S3); however, all 10-ZOL-azole combinations resulted in a reduction in inhibitory dosages, even in the absence of synergy (Table S4 and S5). For both ZOL and 10-ZOL, some antagonism (FICI ≥ 4) was observed with non-azole antifungals, but with the singular exception of 10-ZOL and KET in *Aspergillus frankstonesis*, this was not seen for azole antifungals. To determine whether the spectrum of activity of 10-ZOL extends beyond the fungal kingdom, MICs for 12 pathogenic bacterial species were also performed, but limited or no antibacterial activity was observed (Table S6).

### 10-ZOL is effective in an invertebrate model of infection

**Fig 8:**
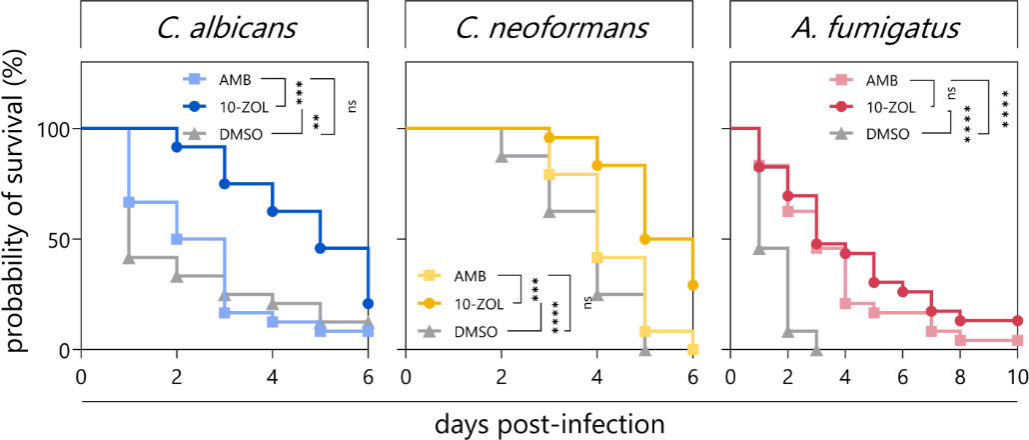
10-ZOL significantly rescues infected *Galleria mellonella* larvae. *Galleria mellonella* larvae (*n* = 24) were infected with *Candida albicans, Cryptococcus neoformans,* or *A. fumigatus*, then treated with a no-drug control (1% DMSO), 10-ZOL at 64 µg/mL, or AMB at 128 µg/mL. Larval mortality was monitored over the course of 10 days. Kaplan-Meier plots represent two independent experiments. Treatments were compared using the log-rank test, **P < 0.01, ***P < 0.001, ****P ≤ 0.0001.

The *in vivo* efficacy of 10-ZOL was evaluated using *Galleria mellonella* larvae infected with *C. albicans*, *C. neoformans,* and *A. fumigatus*. 10-ZOL was non-toxic to *G. mellonella* at concentrations below 128 µg/mL (Fig. S4). Compared to the no-drug control group, a single infusion of 64 µg/mL 10-ZOL significantly reduced mortality in larvae infected with *C. albicans* (*P* = 0.0032), *C. neoformans* (*P* < 0.0001), and *A. fumigatus* (*P* < 0.0001) (Fig. 8). For larvae infected with *A. fumigatus*, there was no significant difference in survival between those treated with 10-ZOL and those treated with 128 µg/mL AMB (*P* = 0.2943). 10-ZOL outperformed AMB in rescuing larvae infected with *C. albicans* (*P* = 0.0004) and *C. neoformans* (*P* = 0.0001).

## DISCUSSION

In this study, we sought to optimize the antifungal activity of FPPS inhibitors by adding lipophilic alkyl tails. These have been shown to improve the bioactivity of FPPS antagonists by enabling coordination with key amino acids in the substrate binding channel (20). The 13 lipophilic prolyl aspartic acids, originally designed to inhibit human FPPS for the treatment of hypercholesterolemia and some cancers (15), were moderately antifungal at best, and many were cytotoxic. In contrast, 10-ZOL, which has previously demonstrated promising antiparasitic activity in protist pathogens (12), was highly active in all fungal species tested. The C1 phosphates that make up bisphosphonates may be essential to their antifungal specificity; both ZOL and 10-ZOL were far more toxic to fungal than to human cells, in contrast to the prolyl aspartic acids that lack these moieties. We therefore propose that 10-ZOL is a promising lead as a novel therapeutic antifungal.

The most remarkable property of 10-ZOL is its spectrum of activity. 10-ZOL was active against all 38 fungal species tested and was overall approximately 100-fold more effective than unmodified ZOL. It was also fungicidal at concentrations near the MIC in several important pathogens, whereas ZOL is largely fungistatic in yeasts (7, 9). 10-ZOL was active in organisms with otherwise limited treatment options, including a pan-resistant strain of *Candida auris* and a highly azole-resistant strain of *Candida glabrata*. Diagnosis and treatment of mycoses are confounded by the diversity of fungal pathogens capable of infecting immunocompromised patients (21, 22), and broad-spectrum antifungals are needed to reduce time-to-treatment in vulnerable patients. Many current-generation and exploratory antifungals like posaconazole (23), ibrexafungerp (24), and olorofim (25) have been described as broad-spectrum but fail to inhibit several important pathogenic taxa, whereas 10-ZOL appears to be active irrespective of species.

Although fewer synergistic interactions were observed between the azole antifungals and 10-ZOL compared to unmodified ZOL, these combinations could still be clinically useful. Combining azoles with 10-ZOL consistently lowered the inhibitory concentrations of both drugs, and this may prevent potentially toxic off-target effects. Increasing the number of therapeutic targets by combining multiple antimicrobials is also known to reduce the development of drug resistance (10, 26), and we have previously shown that this is the case for azole-bisphosphonate combinations in multiple species (7–9).

10-ZOL demonstrated a reasonably wide therapeutic index, meaning that its cytotoxicity in mammalian cells was lower than its antifungal activity (27). In a clinical context, this may reduce the need for intensive drug monitoring and individualization of therapy (28), expediting the resolution of infection. We calculate that the therapeutic index of 10-ZOL is approximately 30 across all pathogens tested; however, it does vary by species. It must be noted that cell line toxicity data fail to account for the less desirable effects of long-term administration of bisphosphonates, which can include osteonecrosis, electrolyte imbalances, and ocular inflammation (29). Our data indicate that 10-ZOL outperforms AMB, a broad-spectrum but highly toxic mainline antifungal (30), in invertebrate models of candidemia and cryptococcosis and performs similarly in a model of aspergillosis despite being administered at a lower dosage. One strategy used to widen the therapeutic index of AMB has been to reformulate it into liposomes (31). 10-ZOL is highly amphiphilic and may be able to spontaneously self-assemble into liposomes (32), so similar approaches could be employed to enhance its bioavailability and reduce host toxicity. This property may also explain the anti-biofilm activity of 10-ZOL, as liposomes improve drug solubility and penetration of the extracellular matrix (33, 34). Furthermore, 10-ZOL has been found to stimulate the release of TNF-α (12), and this is known to induce the proliferation and activation of human γδ T-cells (35), which play a protective role during fungal infection (36). *In vivo*, 10-ZOL may therefore support the immune system to clear fungal growth in infected tissues (37, 38), expanding its therapeutic utility.

The antifungal mechanism of 10-ZOL involves the disruption of the mevalonate pathway and squalene biosynthesis, which is consistent with our previous findings for ZOL (7–9). Squalene deficiency has deleterious effects on membrane integrity, resulting in acute downstream oxidative stress. Curiously, this mechanism differs from the previously reported antiparasitic and anti-cancer mechanisms of lipophilic bisphosphonates, where the inhibition of protein prenylation causes dysregulated cell signaling and mislocalization of membrane proteins (39, 40). It should be noted that the mevalonate pathway is necessary for the synthesis of other vital terpenoids (41), and squalene itself can directly regulate membrane structure (42), so it is possible that bisphosphonates have effects on fungal lipid populations that have not yet been investigated.

The marked increase in the activity of 10-ZOL over ZOL is likely due to increased penetration into the cell facilitated by its lipophilic tail, rather than changes to FPPS binding. Although alkylating bisphosphonates improved binding in human FPPS (20), this did not affect the binding or activity of geranylgeranyl diphosphate synthase (GGDPS) in *Plasmodium falciparum,* despite a marked increase in antiparasitic activity (13). More broadly, it has been reported that there is only a weak correlation between bisphosphonate-enzyme binding affinity and anti-*Plasmodium* activity (43). Amphiphilic biologics like 10-ZOL are often able to incorporate themselves into phospholipid bilayers and compromise membrane integrity (44), and this may benefit from the membrane-disruptive effects of squalene and ergosterol depletion due to inhibited FPPS, resulting in increased fungal eradication.

### Conclusion

Escalating infection rates and resistance to mainline antifungals require the urgent development of new antifungals and an exploration of novel druggable targets. Building on a body of research that validates the inhibition of fungal FPPS and the mevalonate pathway as a chemotherapeutic strategy, we show promising inhibition by 10-ZOL, an analog of ZOL with a 10-carbon lipophilic tail. The high *in vitro* and *in vivo* antifungal efficacy of 10-ZOL and its low toxicity in mammalian cells make 10-ZOL a promising candidate for future clinical translation. Our work validates rational drug design, as the initial identification of fungal FPPS as a therapeutic target by proteome analysis, followed by the analysis of various FPPS inhibitors and structural optimization, allowed us to systematically identify a potent antifungal lead compound.

## MATERIALS AND METHODS

### Fungal pathogens

Thirty-eight species of clinically relevant fungi were used in this study. The source of each isolate is detailed in Table S3. All isolates were maintained on Sabouraud’s dextrose agar (SDA), except for *Trichophyton rubrum*, which was grown on Oatmeal Agar to improve conidial yield. For mechanistic studies, experiments were performed on *C. albicans* SC5314, *C. neoformans* H99, and *A. fumigatus* ATCC 204305.

### Antifungal agents and FPPS inhibitors

Stock solutions of ZOL (Sapphire Bioscience) were prepared in 0.1 N NaOH at 12.8 mg/mL. Antifungals FLC, ITR, KET, AMB (Sapphire Bioscience), caspofungin (CAS), and 5-flucytosine (5FC) (Sigma Aldrich) were prepared according to the CLSI standard M27-Ed4 for antifungal susceptibility testing (16).

10-ZOL was synthesized as described previously (12), with modifications detailed in the supplemental methods. The synthesis of all prolyl-aspartic acid derivatives was performed as previously described (15) and is outlined in the supplemental methods. Stock solutions of all FPPS inhibitors were prepared at 12.8 mg/mL in DMSO.

The drug solutions used in all mechanistic experiments were standardized to 1% DMSO, and 1% DMSO was used as a no-drug solvent control throughout this study.

### Inhibition assays

Antifungal susceptibility was determined by broth microdilution according to the CLSI guidelines described in M27-Ed4 (16). Maximum test concentrations were 128 mg/L for FLC, ZOL, 10-ZOL, and all prolyl-aspartate derivatives, 16 mg/L for ITR, VOR, KET, and AMB, and 64 mg/L for 5FC. MIC_80_ was read for azole antifungals, MIC_50_ was read for CAS, and 5FC and MIC_100_ were read for all other compounds. MFC was determined by back-plating drug-treated cultures from MIC broths onto SDA plates. The MFC was defined as the lowest concentration from which no colonies could be cultured.

Drug interactions between antifungals and FPPS inhibitors were investigated using a simplified checkerboard diagonal screening method (45). Interaction results were assessed visually at 80% inhibition for combinations containing CAS and 100% inhibition for all other combinations. FICIs were determined according to the Loewe additivity model (17).

The inhibitory activity of 10-ZOL and azole antifungals against fungal biofilms was investigated using the XTT reduction assay (46). Biofilms of *Cryptococcus* were seeded in DMEM, and all other species were seeded in RPMI-1640. The maximum concentrations tested were 2,048 mg/L for FLC and 256 mg/L for ITR, KET, and 10-ZOL. SIC_80_ was determined as the concentration where metabolic activity decreased by 80% compared to untreated biofilms. To investigate interactions between 10-ZOL and azoles, fungal biofilms were treated with the two agents in a checkerboard assay, and the SFICI was calculated.

### Cytotoxicity assays

The cytotoxicity of all FPPS inhibitors in HEK-293T cells was determined by MTT reduction assay (47). Cells were treated for 72 h with a serial dilution of the drug in DMEM (Sigma Aldrich) at a maximum concentration of 256 mg/L. Non-linear regression analysis was performed to obtain dose-response curves and calculate the IC_50_.

### Squalene rescue assay

Squalene rescue assays were performed as described previously (7). Serial twofold dilutions of squalene (Sigma Aldrich) starting at 256 mg/L were added to *C. albicans*, *C. neoformans,* and *A. fumigatus* inocula prepared according to the CLSI guidelines described above, with and without 10-ZOL at 1× MIC. After incubation at 35°C for 24 h (*C. albicans*), 48 h (*A. fumigatus*), or 72 h (*C. neoformans*), OD_600_ was measured in a BioTek ELx800 plate reader.

### Ergosterol quantitation

Overnight cultures of *C. albicans* and *C. neoformans* were adjusted to 10^5^ cells/mL and *A. fumigatus* conidia were harvested and adjusted to 10^4^ spores/mL in 10 mL yeast peptone dextrose (YPD) containing 1% DMSO (no-drug solvent control), FLC at 1× MIC, or 10-ZOL at 0.25, 1, or 4× MIC. All treatments were either supplemented with 128 mg/L squalene or included 0.1% acetone as a no-squalene control. Treated cultures were incubated at 30°C with shaking for 18 h for *C. albicans* and *A. fumigatus* and for 32 h for *C. neoformans*. Cell membrane ergosterol was then extracted and quantified as described previously (48).

### Time-kill assays

Time-kill assays were performed to determine the fungicidal kinetics of 10-ZOL. Cultures of 10^5^ cells of *Candida albicans* or *Cryptococcus neoformans* were prepared in 10 mL of YPD broth containing 1% DMSO (no-drug solvent control) and 10-ZOL at 0.25, 0.5, 1, 2, and 4× MIC. Cultures were incubated at 37°C with shaking at 200 rpm. Aliquots were withdrawn at 12, 24, 36, and 48 h post-treatment for both species, and additionally at 60 and 72 h for *C. neoformans*, then back-plated onto SDA. Viable cell counts were obtained after 48 h.

### Membrane integrity assays

The effect of 10-ZOL on membrane fluidity was determined using TMA-DPH steady-state anisotropy (49). Overnight cultures of *C. albicans* and *C. neoformans* were adjusted to 10^5^ cells/mL in 2 mL YPD broth containing 1% DMSO (no-drug solvent control) or 10-ZOL at 0.25, 0.5, 1, 2, or 4× MIC. *Candida albicans* cells were incubated for 6 h, and *C. neoformans* cells were incubated for 9 h at 30°C, washed, then labeled with 2 µM TMA-DPH in PBS for 10 min. For *A. fumigatus*, 10^6^ conidia were harvested and cultured in 2 mL YPD containing 10-ZOL, then incubated overnight at 30°C at 250 rpm. Mycelia were fixed, and protoplasts were prepared as described previously (50). Protoplasts were washed twice and labeled with 2 µM TMA-DPH in PBS for 30 min. For all labeled cultures, parallel and perpendicular fluorescence intensities were measured at 440 nm in a CLARIOstar plate reader (BMG Labtech), and anisotropy was calculated.

Membrane depolarization was determined using potential-sensitive fluorophore diS-C_3_(3) (Sigma), as described previously (51). Yeast cells and *A. fumigatus* conidia were treated as described above, then washed with citrate phosphate buffer and labeled with 20 nM diS-C_3_(3) at a final concentration of 10^6^ cells/mL for yeasts and 10^4^ conidia/mL for *Aspergillus*. Cells were transferred to a microplate (Invitrogen), then emission spectra between 560 and 585 nm (λ_excitation_ = 531 nm) were obtained every 12 min for 1 h using a CLARIOstar plate reader (BMG Labtech). The emission λ_max_ was determined at each timepoint.

Membrane permeabilization was determined by measuring the leakage of nucleic acid into the supernatant (19). For *C. neoformans* and *C. albicans*, cells from overnight cultures were adjusted to 10^6^ cells/mL in 2 mL of Milli-Q water containing 1% DMSO or 10-ZOL at 0.25–4× MIC. For *A. fumigatus*, conidia from fresh cultures were harvested and adjusted to 10^4^ spores/mL in Milli-Q water containing 1% DMSO or 10-ZOL at 0.25–4× MIC. Suspensions were incubated at 30°C with shaking at 200 rpm for 6 h, then passed through a 0.45 µm syringe filter. The A_260_ of each filtrate was measured with a DS-11 FX +benchtop spectrophotometer (DeNovix).

### Oxidative stress assays

ROS accumulation in 10-ZOL-treated fungi was investigated using DCFDA (Sigma Aldrich), a ROS-sensitive fluorophore (18). Yeast cells from overnight cultures were adjusted to 10^5^ cells/mL in 2 mL YPD containing 0.25–4× MIC 10-ZOL or 1% DMSO. Conidia of *A. fumigatus* were harvested and adjusted to 10^4^ conidia/mL in 2 mL YPD containing 0.25–4× MIC 10-ZOL or 1% DMSO. All cultures were incubated for 6 h and then labeled with 20 µM DCFDA in PBS for 30 min. Labeled cultures were transferred into a microplate, and fluorescence intensity was measured using a CLARIOstar plate reader (BMG Labtech).

Oxidative damage in 10-ZOL-treated cells was investigated using a TBARS assay, which measures peroxidation of lipids by free oxygen radicals (52). Yeasts from overnight cultures were pelleted, washed, and resuspended in 10 mL YPD containing 0.25–4 × MIC 10-ZOL or 1% DMSO. *Aspergillus fumigatus* conidia were adjusted to 10^5^ spores/mL in 10 mL YPD with 0.25–4 × MIC 10-ZOL. Cultures were incubated overnight, pelleted, and resuspended in 1 mL ice-cold PBS. Malondialdehyde (MDA) was extracted and quantified using a TBARS assay kit (Cayman Chemical #700870) according to the manufacturer’s instructions. MDA was normalized to total protein content in cell lysates.

### *Galleria mellonella* survival assays

*Galleria mellonella* larvae were reared and prepared as described previously (53). Larvae (*n* = 12 per treatment) were each inoculated with 10^5^ spores or cells of *A. fumigatus*, *C. albicans,* or *C. neoformans* in 10 µL of PBS. After 2 h of recovery, 10 µL of 1% DMSO, 64 µg/mL 10-ZOL, or 128 µg/mL AMB were injected into the larval hemolymph via the last-left proleg. Treated larvae were incubated at 37°C, and mortality was evaluated every day for 10 days.

### Statistical analyses

All experiments were conducted using three biological replicates, each with two technical replicates unless otherwise indicated. MICs and FICIs were compared by one-way ANOVA. Squalene-dependent rescue of ergosterol biosynthesis was determined by Welch’s *t*-test. Time-kill curves, anisotropy, membrane leakage, ROS accumulation, and lipid peroxidation were compared by one-way ANOVA. Dose-dependent effects and correlations between 10-ZOL MIC and squalene rescue EC_50_ were determined by calculating the Pearson correlation coefficient, *r. Galleria mellonella* survival curves were compared using the log-rank *P*-test.

## SUPPLEMENTAL MATERIAL

Supplemental figures mbio.03199-25-s0001.pdf Figures S1-S4.

Supplemental text mbio.03199-25-s0002.pdf Supplemental methods.

Supplemental tables mbio.03199-25-s0003.xlsx Tables S1-S6.

ASM does not own the copyrights to Supplemental Material that may be linked to, or accessed through, an article. The authors have granted ASM a non-exclusive, world-wide license to publish the Supplemental Material files. Please contact the corresponding author directly for reuse.
