# Supplementary material for: Alkylation-based optimization of antifungal FPPS inhibitors yields a potent, broad-spectrum lipophilic zoledronate derivative
Source: mBio. 2025 Dec 22;17(2):e03199-25. doi: 10.1128/mbio.03199-25 (PMC12892976; doi:10.1128/mbio.03199-25)
Supplement: Supplemental figures — Figures S1-S4. [file mbio.03199-25-s0001.pdf]

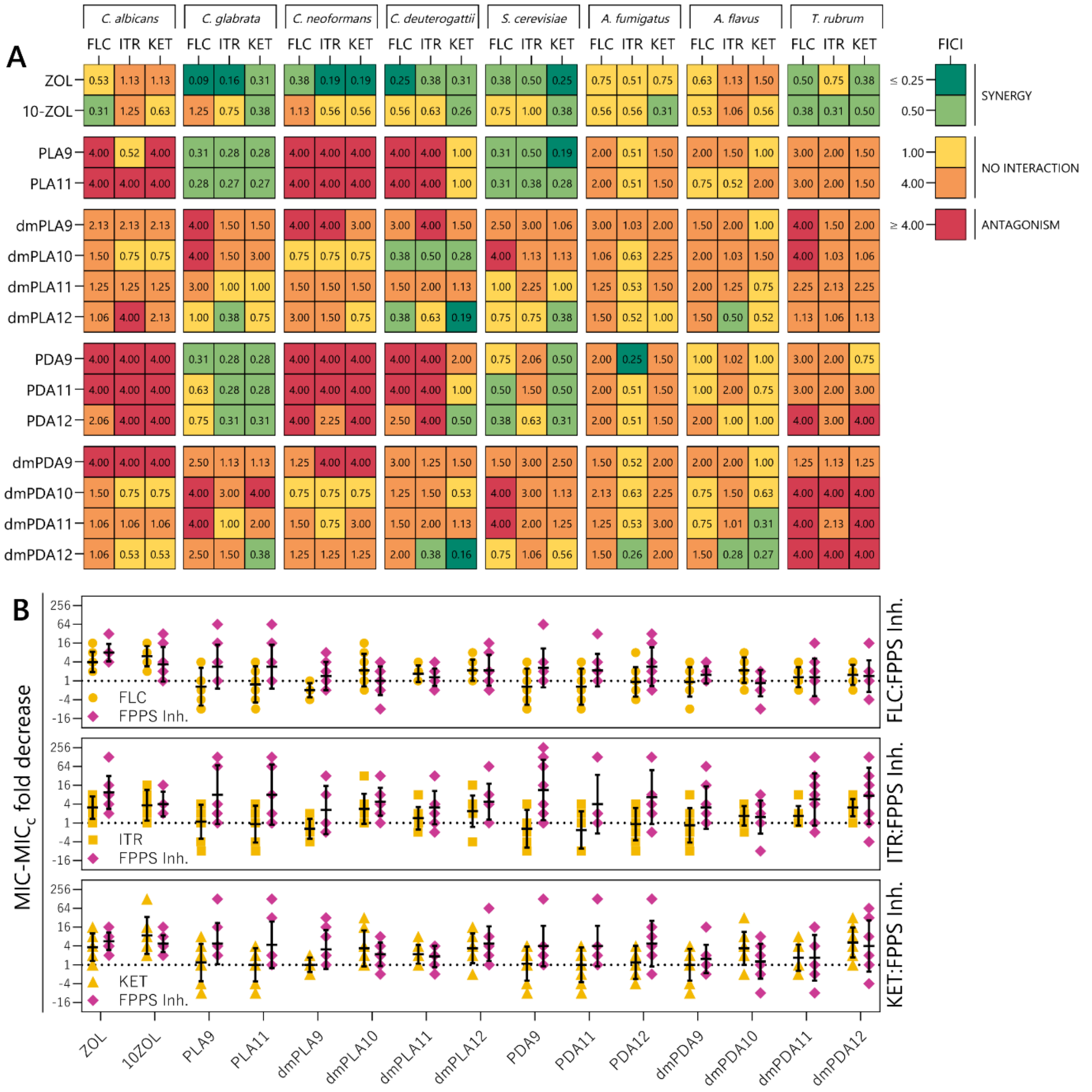

Supplementary Figure S1. Lipophilic FPPS inhibitors vary in their interactions with azole antifungals. (A) Heatmaps displaying the FICI values for ZOL, 10-ZOL and other lipophilic FPPS inhibitors when combined with azole antifungals fluconazole (FLC), itraconazole (ITR), and ketoconazole (KET) against various fungal pathogens. FICIs are the means of three biological replicates. (B) Fold-decrease in MIC for each azole and FPPS inhibitor when used in combination compared to when used alone. Each point represents the fold-decrease in a representative of each fungal species tested, and positive values indicate a reduction in effective dosages. MICs, MIC$_c$s and fold-decreases are detailed in Supplementary Table 1.

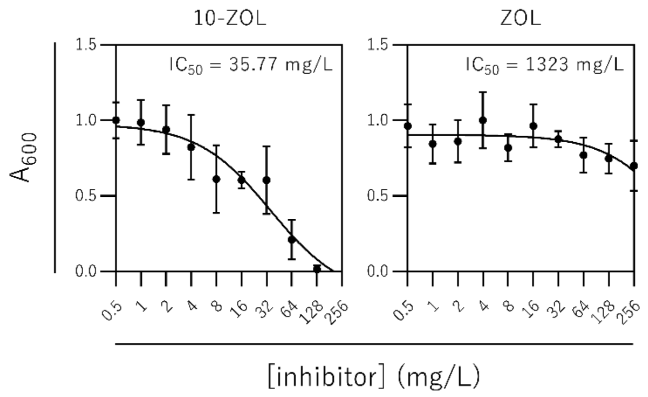

Supplementary Figure S2. Cytotoxicity of zoledronate and 10-ZOL in HEK-293t cells.

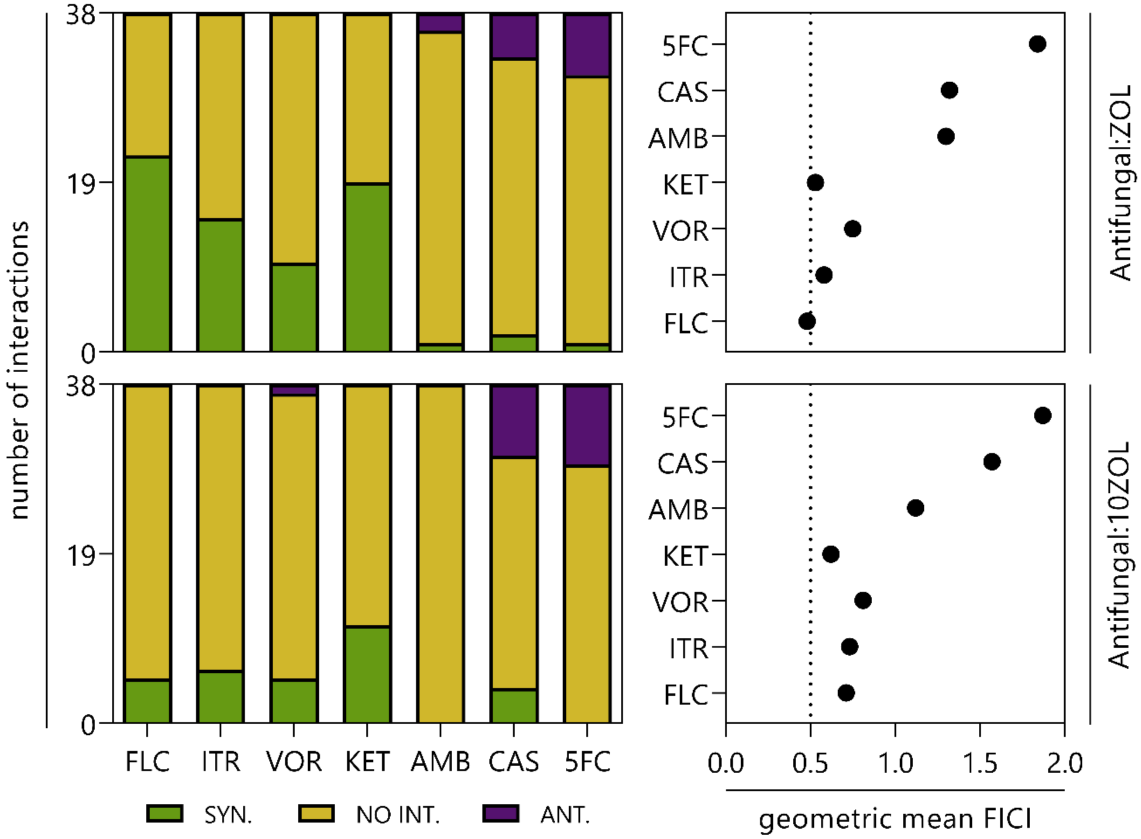

Supplementary Figure S3. Number of antifungal-bisphosphonate pairings that are synergistic, antagonistic, or have no interaction (left) and their geometric mean FICIs (right).

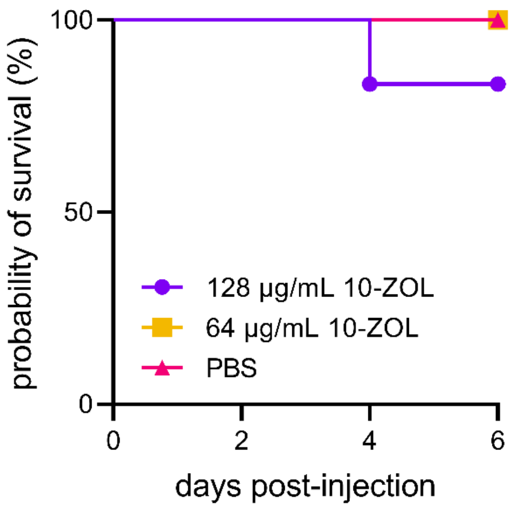

Supplementary Figure S4. 10-ZOL is non-toxic to *Galleria mellonella* at 64 µg/mL. Results are for one biological replicate (n = 6 larvae per treatment).
