# Supplementary material for: Alkylation-based optimization of antifungal FPPS inhibitors yields a potent, broad-spectrum lipophilic zoledronate derivative
Source: mBio. 2025 Dec 22;17(2):e03199-25. doi: 10.1128/mbio.03199-25 (PMC12892976; doi:10.1128/mbio.03199-25)
Supplement: Supplemental text — Supplemental methods. [file mbio.03199-25-s0002.pdf]

Supplementary Methods

Synthesis of lipophilic L-prolyl-aspartic acid derivatives

A series of compounds were synthesised based on the chemical scaffold of dipeptide **5**, with different enantiomers of aspartic acids (D and L), as well as different modifications at the R^1, R^2 and R^3 positions (**Scheme 1**).

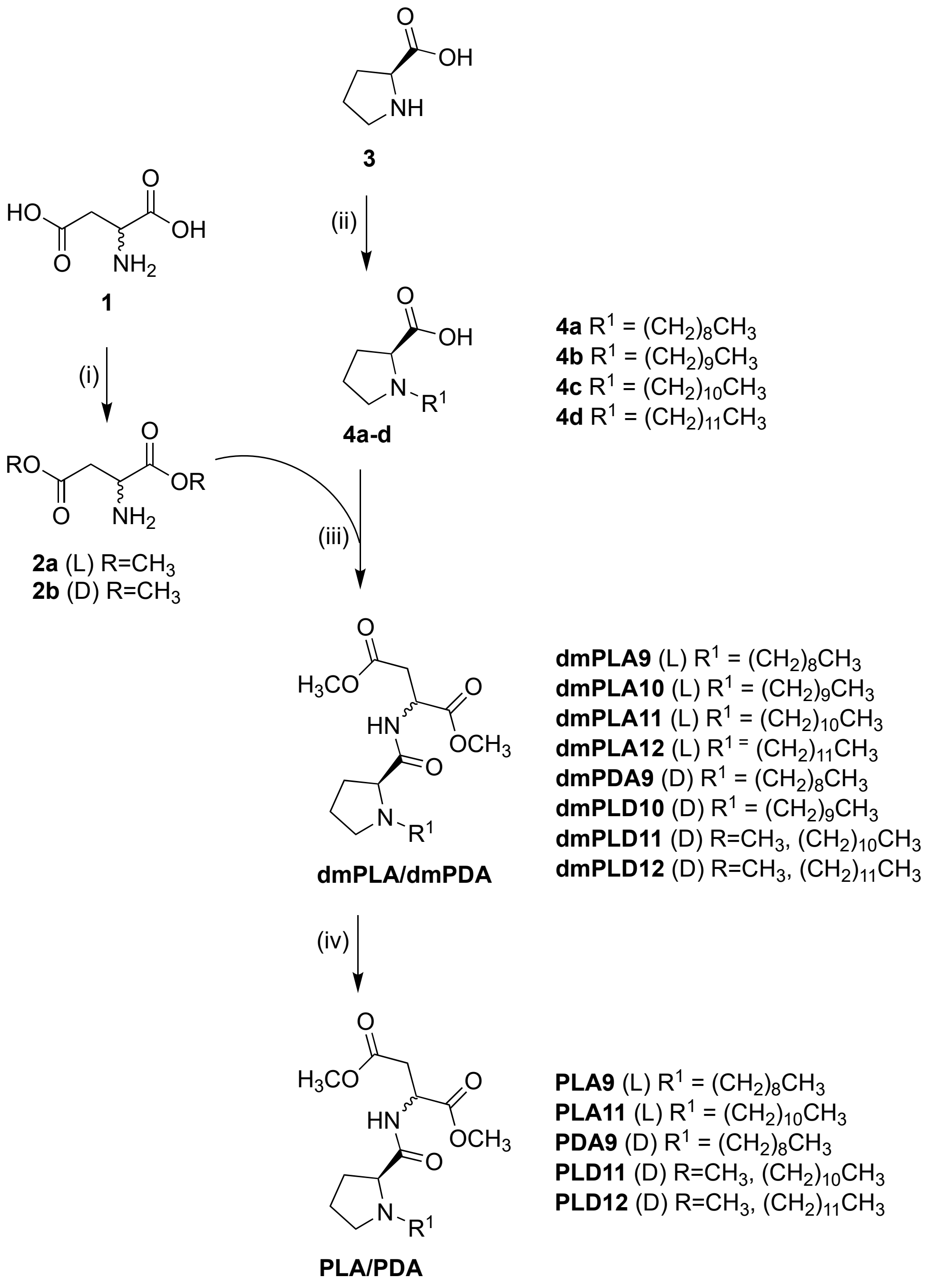

Scheme 1. Synthesis of amides **7** and **8**. Reagents and conditions: (i) $SOCl_2$ (3 equiv.), MeOH, 0 °C to rt, 24 h; (ii) alkyl bromide (1.5 equiv.), K_2CO_3 (2 equiv.), MeOH, 75 °C, 48 h; (iii) IBCF (1.1 equiv.), DIPEA (1.1 equiv.), anhyd. DCM, 0 °C to rt, 24 h (iv) LiOH (4 equiv.) MeOH/H_2O, 100 °C, 2 h.

The synthetic route shown in **Scheme 1** begins with the preparation of the aminoesters **2a & b** using Methyl ester **1a** was synthesised from L-aspartic acid and **1b** from D-aspartic acid in methanol. Nucleophilic substitution of an alkyl bromide with L-proline **3** gave the *N*-alkyl prolines **4a-d**. Coupling of aminoesters **2a & b** with *N*-alkyl prolines **4a-d** gave **dmPLA9-12** and **dmPLD9-12**, and subsequent hydrolysis to afforded **- PLA9, PLA11, PDA9, PDA11** and **PDA12**.

General procedure for esterification of aspartic acid 1a & b

D/L-Aspartic acid (1 equiv.) was suspended in alcohol and stirred at 0 °C, then thionyl chloride (3 equiv.) was added dropwise to the reaction mixture. The reaction was warmed to room temperature after 15 minutes and left to stir at room temperature overnight. After removal of the solvent *in vacuo*, the residue was dissolved in DCM (2 mL) and the volatiles were removed *in vacuo*. The process was repeated until the crude product turned into a white powder.

L-Aspartic acid dimethyl ester 1a

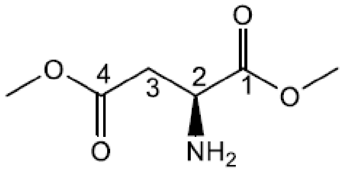

Dimethyl ester **1a** was synthesised from L-aspartic acid (0.2 g, 1.5 mmol) and thionyl chloride (0.54 g, 4.5 mmol) in methanol (25 mL) using the method above and was obtained as a white solid (0.28 g, 97%); ^{1}H NMR (400 MHz, CD_3OD) δ_H 3.07 (2H, d, J = 5.3 Hz, CH_2), 3.75 (3H, s, CH_3O), 3.84 (3H, s, CH_3O), 4.40 (1H, t, J = 5.4 Hz, CH); ^{13}C NMR (100 MHz, CH_3OD) δ_C 34.8 (CH_2, C-3), 50.4 (CH, C-2), 53.0 (CH_3O), 54.0 (CH_3O), 169.6 (C=O), 171.4 (C=O); LRMS (ESI), *m/z* 162 $[M + H]^+$.

D-Aspartic acid dimethyl ester 1b

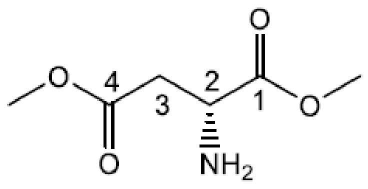

Dimethyl ester **1b** was synthesised from D-aspartic acid (0.5 g, 3.8 mmol) and thionyl chloride (1.34 g, 11.3 mmol) in methanol (50 mL) using the method above and was obtained as a white solid (0.68 g, 95%); ^{1}H NMR (400 MHz, CD_3OD) δ_H 3.06 (2H, d, J = 5.4 Hz, CH_2), 3.76 (3H, s, CH_3O), 3.85 (3H, s, CH_3O), 4.40 (1H, t, J = 5.4 Hz, CH); ^{13}C NMR (100 MHz, CH_3OD) δ_C 34.9 (CH_2, C-3), 50.5 (CH, C-2), 53.2 (CH_3O), 54.2 (CH_3O), 169.6 (C=O), 171.4 (C=O); LRMS (ESI), *m/z*: 162 $[M + H]^+$.

General procedure for the synthesis of N-alkyl-L-proline 4a-d

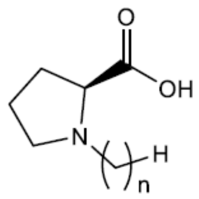

A mixture of L-proline (1 equiv.), alkyl bromide (1.5 equiv.) and K_2CO_3 (2 equiv.) in methanol was refluxed for 48 hours. Upon filtration and removal of solvent *in vacuo*, the crude product was taken up in water and the pH was adjusted to 4 with 6M HCl. The aqueous layer was then extracted with chloroform 5 times. The combined organic layers were dried over Na_2SO_4 and reduced *in vacuo* and the crude was purified by column chromatography on silica, eluting with ethyl acetate: methanol (70:30 to 50:50) and gave products of a moist and viscous texture. The compounds were further purified by recrystallisation in water/acetone to give dry N-alkyl-L-proline crystals.

Nonyl-L-proline 4a

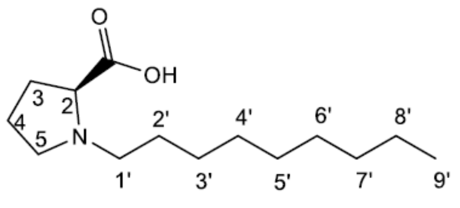

Nonyl-L-proline was synthesised from L-proline (2 g, 17.4 mmol) and 1-bromononane (5.4 g, 26.1 mmol) with potassium carbonate (4.8 g, 34.8 mmol) in methanol (50 mL) using the method above and was obtained as a white solid (3.66 g, 87%); ^{1}H NMR (400 MHz, CD_3OD) δ_H 0.90 (3H, t, J = 6.8 Hz, CH_3-9’), 1.24-1.44 (12H, m, CH_2-3’, CH_2-4’, CH_2-5’, CH_2-6’, CH_2-7’, CH_2-8’), 1.71 (2H, m, CH_2-2’), 1.92 (1H, m, CH_AH_B-3), 2.11 (2H, CH_2-4), 2.42 (1H, m, CH_AH_B-3), 3.09 (2H, m, CH_2-1’), 3.22 (1H, m,

CH_AH_B-5), 3.72 (1H, m, CH_AH_B-5), 3.83 (1H, m, CH-2); ^{13}C NMR (100 MHz, CH3OD) δ_C 14.4 (CH_3, C-9'), 23.7 (CH_2, C-8'), 24.4 (CH_2, C-4), 26.9 (CH_2, C-2'), 27.6 (CH_2), 30.2 (CH_2), 30.3 (CH_2), 30.3 (CH_2), 30.5 (CH_2), 33.0 (CH_2), 56.0 (CH_2, NCH_2), 56.8 (CH_2, NCH2), 70.7 (CH, C-2), 173.4 (C=O); LRMS (ESI), *m/z*: 242 $[M + H]^+$.

Decyl-L-proline 4b

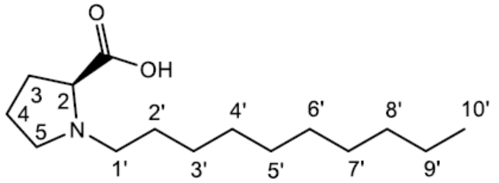

Decyl-L-proline was synthesised from L-proline (1 g, 8.7 mmol) and 1-bromodecane (2.89 g, 13.1 mmol) with potassium carbonate (2.4 g, 17.4 mmol) in methanol (50 mL) using the method above and was obtained as a white solid (1.90 g, 86%); ^{1}H NMR (400 MHz, CD_3OD) δ_H 0.90 (3H, t, J = 6.8 Hz, CH_3-10'), 1.24-1.46 (14H, m, CH_2-3', CH_2-4', CH_2-5', CH_2-6', CH_2-7', CH_2-8', CH_2-9'), 1.72 (2H, m, CH_2-2'), 1.97 (1H, m, CH_AH_B-3), 2.12 (2H, CH_2-4), 2.44 (1H, m, CH_AH_B-3), 3.10 (2H, m, CH_2-1'), 3.25 (1H, m, CH_AH_B-5), 3.74 (1H, m, CH_AH_B-5), 3.91 (1H, m, CH-2); ^{13}C NMR (100 MHz, CH3OD) δ_C 14.4 (CH_3, C-10'), 23.7 (CH_2, C-9'), 24.3 (CH_2, C-4), 26.9 (CH_2, C-2'), 27.6 (CH_2), 30.2 (CH_2), 30.2 (CH_2), 30.4 (CH_2), 30.5 (CH_2), 30.6 (CH_2), 33.0 (CH_2), 56.0 (CH_2, NCH_2), 56.8 (CH_2, NCH_2), 70.2 (CH, C-2), 173.1 (C=O); LRMS (ESI), *m/z*: 256 $[M + H]^+$.

Undecyl-L-proline 4c

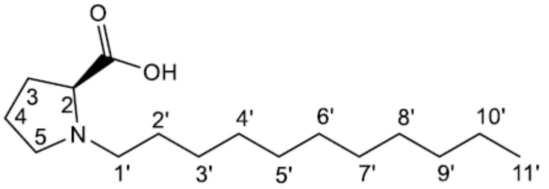

Undecyl-L-proline was synthesised from L-proline (2 g, 17.4 mmol) and 1-bromoundecane (6.13 g, 26.1 mmol) with potassium carbonate (4.8 g, 34.8 mmol) using the method above and was obtained as a white solid (3.07 g, 65%); ^{1}H NMR (400 MHz, CD_3OD) δ_H 0.90 (3H, t, J = 6.8 Hz, CH_3-11'), 1.23-1.45 (16H, m, CH_2-3', CH_2-4', CH_2-5', CH_2-6', CH_2-7', CH_2-8', CH_2-9', CH_2-10'), 1.71 (2H, m, CH_2-2'), 1.95 (1H, m, CH_AH_B-3), 2.10 (2H, CH_2-4), 2.42 (1H, m, CH_AH_B-3), 3.08 (2H, m, CH_2-1'), 3.26 1(1H, m, CH_AH_B-5), 3.72 (1H, m, CH_AH_B-5), 3.85 (1H, m, CH-2); ^{13}C NMR (100 MHz, CH_3OD) δ_C 14.4 (CH_3, C-12'), 23.7 (CH_2, C-11'), 24.4 (CH_2, C-4), 27.0 (CH_2, C-2'), 27.6 (CH_2), 30.2 (CH_2), 30.3 (CH_2), 30.5 (CH_2), 30.5 (CH_2), 30.7 (CH_2), 30.7 (CH_2), 33.1 (CH_2), 56.0 (CH_2, NCH_2), 56.8 (CH_2, NCH_2), 70.7 (CH, C-2), 173.5 (C=O); LRMS (ESI), *m/z*: 270 $[M + H]^+$.

Dodecyl-L-proline 4d

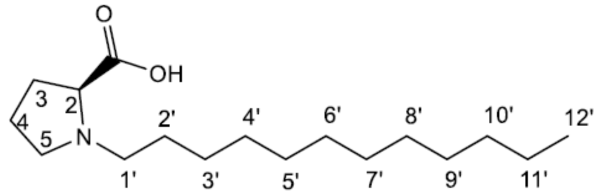

Dodecyl-L-proline was synthesised from L-proline (2 g, 17.4 mmol) and 1-bromododecane (6.49 g, 26.1 mmol) with potassium carbonate (4.8 g, 34.8 mmol) using the method above and was obtained as a white solid (3.90 g, 79%); ^{1}H NMR (400 MHz, CD_3OD) δ_H 0.90 (3H, t, J = 7.0 Hz, CH_3-12'), 1.24-1.44 (18H, m, CH_2-3', CH_2-4', CH_2-5', CH_2-6', CH_2-7', CH_2-8', CH_2-9', CH_2-10', CH_2-11'), 1.72 (2H, m, CH_2-2'), 1.99 (1H, m, CH_AH_B-3), 2.14 (2H, CH_2-4), 2.48 (1H, m, CH_AH_B-3), 3.13 (2H, m, CH_2-1'), 3.26 (1H, m, CH_AH_B-5), 3.73 (1H, m, CH_AH_B-5), 4.03 (1H, m, CH-2); ^{13}C NMR (100 MHz, CH_3OD) δ_C 14.4 (CH_3, C-12'), 23.7 (CH_2, C-11'), 24.1 (CH_2, C-4), 26.9 (CH_2, C-2'), 27.6 (CH_2), 30.0 (CH_2), 30.2 (CH_2), 30.5 (CH_2), 30.5 (CH_2), 30.6 (CH_2), 30.7 (CH_2), 33.1 (CH_2), 56.1 (CH_2, NCH_2), 56.9 (CH_2, NCH_2), 69.7 (CH, C-2), 172.5 (C=O); LRMS (ESI), *m/z*: 284 $[M + H]^+$.

General procedure for the coupling reaction to give I dmPLA9-12 and dmPDA9-12

Isobutyl chloroformate (1.1 equiv.) was dissolved in anhydrous DCM and left to stir at 0 °C. A mixture of acid (1 equiv.) and DIPEA (1.1 equiv.) in anhydrous DCM was added dropwise to the reaction mixture over 30 minutes. After 30 minutes, a solution of amine in anhydrous DCM was added to the reaction mixture dropwise over 30 minutes. The mixture was allowed to warm to room temperature and left to stir overnight. Upon completion of reaction, the solvent is removed in vacuo, the crude product was taken up in ethyl acetate and washed with 10% citric acid (2 x 5mL). The combined organic layer was dried over anhydrous Na_2SO_4 and reduced in vacuo and the crude was purified by flash column chromatography on silica.

Dimethyl nonyl-L-prolyl-L-aspartate dmPLA9

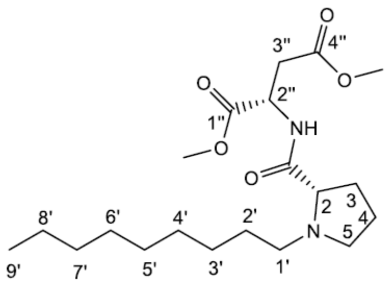

Dimethyl nonyl-L-prolyl-L-aspartate was synthesised from nonyl-L-proline (1 g, 4.2 mmol) and L-aspartate dimethyl ester (0.67 g, 4.2 mmol) with isobutyl chloroformate (0.63 g, 4.6 mmol) and DIPEA (0.6 g, 4.6 mmol) using the method described above. The crude product was purified by flash column chromatography on silica eluting with ethyl acetate: methanol (75:25 to 0:100), and the final product was obtained as a brown oil (0.90 g, 56%); FTIR (neat, v/cm-1): 3209 (NH, amide), 1734 (C=O, ester), 1681 (C=O, amide); 1172 (C-O, ester), 1001 (C-O, ester); ^{1}H NMR (400 MHz, CD_3OD) δ_H 0.90 (3H, t, J = 6.8 Hz, CH_3-9'), 1.31-1.36 (12H, m, CH_2-3', CH_2-4', CH_2-5', CH_2-6', CH_2-7', CH_2-8'), 1.67 (2H, m, CH_2-2'), 1.96-2.26 (3H, m, CH_2-4, $C\mathit{H}_AH_B$-3), 2.58 (1H, m, $CH_A\mathit{H}_B$-3), 2.90 (1H, dd, J = 16.8 and 7.4 Hz, $C\mathit{H}_AH_B$-3''), 2.97 (1H, dd, J = 16.8 and 4.9 Hz, $CH_A\mathit{H}_B$-3''), 3.11-3.28 (3H, m, CH_2-1' and $C\mathit{H}_AH_B$-5), 3.70 (3H, s, CH_3O), 3.74 (3H, s, CH_3O), 3.74-3.81 (1H, m, $CH_A\mathit{H}_B$-5), 4.19 (1H, m, CH-2), 4.82 (1H, m, CH-2''); ^{13}C NMR (100 MHz, CH_3OD) δ_C 14.4 (CH_3, C-9'), 23.7 (CH_2, C-8'), 24.0 (CH_2, C-4), 26.8 (CH_2, C-2'), 27.5 (CH_2), 30.1 (CH_2), 30.3 (CH_2), 30.4 (CH_2), 33.0 (CH_2), 36.4 (CH_2), 36.5 (CH_2, C-3''), 50.7 (CH, C-2''), 52.5 (CH_3O), 53.3 (CH_3O), 56.3 (CH_2, NCH_2), 56.6 (CH_2, NCH_2), 68.5 (CH, C-2), 169.2 (C=O, NH*C*O), 172.3 (C=O), 172.9 (C=O); LRMS (ESI), *m/z*: 385 $[M + H]^+$; (ESI/FT-MS/+ve) m/z: $[M + H]^+$ Calcd for $C_{20}H_{36}N_2O_5$ 385.2697; Found 385.2697.

Dimethyl decyl-L-prolyl-L-aspartate dmPLA10

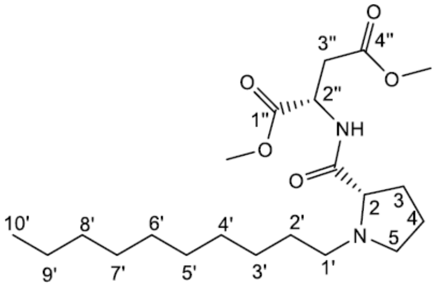

Dimethyl decyl-L-prolyl-L-aspartate was synthesised from decyl-L-proline (0.79 g, 3.1 mmol) and L-aspartate dimethyl ester (0.5 g, 3.1 mmol) with isobutyl chloroformate (0.47 g, 3.4 mmol) and DIPEA (0.44 g, 3.4 mmol) using the method described above. The crude product was purified by flash column chromatography on silica eluting with ethyl acetate: methanol (75:25 to 0:100), and the final product was obtained as a brown oil (0.54 g, 44%); FTIR (neat, v/cm-1): 3199 (NH, amide), 1739 (C=O, ester), 1681 (C=O, amide), 1170 (C-O, ester), 1002 (C-O, ester); ^{1}H NMR (400 MHz, CD_3OD) δ_H 0.90 (3H, t, J = 6.9 Hz, CH_3-10'), 1.31-1.36 (14H, m, CH_2-3', CH_2-4', CH_2-5', CH_2-6', CH_2-7', CH_2-8', CH_2-9'), 1.67 (2H, m, CH_2-2'), 2.02-2.19 (3H, m, CH_2-4, $C\mathit{H}_AH_B$-3), 2.57 (1H, m, $CH_A\mathit{H}_B$-3), 2.90 (1H, dd, J = 16.8 and 7.4 Hz, $C\mathit{H}_AH_B$-3''), 2.97 (1H, dd, J = 16.8 and 4.9 Hz, $CH_A\mathit{H}_B$-3''), 3.11-3.27 (3H, m, CH_2-1' and $C\mathit{H}_AH_B$-5), 3.70 (3H, s, CH_3O), 3.74 (3H, s, CH_3O), 3.74-3.80 (1H, m, $CH_A\mathit{H}_B$-5), 4.17 (1H, m, CH-2), 4.85 (1H, m, CH-2''); ^{13}C NMR (100 MHz, CH_3OD) δ_C 14.2 (CH_3, C-10'), 23.7 (CH_2, C-9'), 24.0 (CH_2, C-4), 26.8

(CH_2, C-2'), 27.6 (CH_2), 30.2 (CH_2), 30.4 (CH_2), 30.5 (CH_2), 30.6 (CH_2), 33.0 (CH_2, C-8'), 36.4 (CH_2, C-3''), 50.7 (CH, C-2''), 52.6 (CH_3O), 53.2 (CH_3O), 56.3 (CH_2, NCH_2), 56.6 (CH_2, NCH_2), 68.6 (CH, C-2), 169.3 (C=O, NH*C*O), 171.8 (C=O), 172.1 (C=O); LRMS (ESI), *m/z*: 399 $[M + H]^+$; HRMS (ESI/FT-MS/+ve) m/z: $[M + H]^+$ Calcd for $C_{21}H_{38}N_2O_5$ 399.2853; Found 399.2854.

Dimethyl undecyl-L-prolyl-L-aspartate dmPLA11

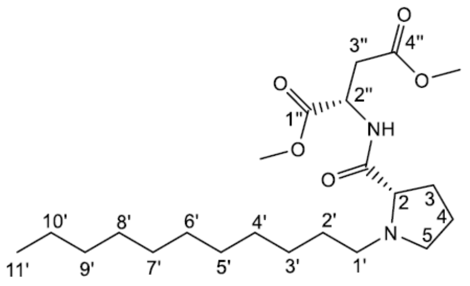

Dimethyl undecyl-L-prolyl-L-aspartate was synthesised from undecyl-L-proline (1 g, 3.7 mmol) and L-aspartate dimethyl ester (0.6 g, 3.7 mmol) with isobutyl chloroformate (0.56 g, 4.1 mmol) and DIPEA (0.53 g, 4.1 mmol) using the method described above. The crude product was purified by flash column chromatography on silica eluting with ethyl acetate: methanol (75:25 to 0:100), and the final product was obtained as a brown oil (0.65 g, 42%); FTIR (neat, v/cm-1): 3209 (NH, amide), 1739 (C=O, ester), 1681 (C=O, amide), 1172 (C-O, ester), 1002 (C-O, ester); ^1H NMR (400 MHz, CD_3OD) δ_H: 0.90 (3H, t, J = 6.9 Hz, CH_3-11') , 1.30-1.36 (16H, m, CH_2-3', CH_2-4', CH_2-5', CH_2-6', CH_2-7', CH_2-8', CH_2-9', CH_2-10'), 1.67 (2H, m, CH_2-2'), 2.03-2.18 (3H, m, CH_2-4, C$H_A$$H_B$-3), 2.57 (1H, m, C$H_A$$H_B$-3), 2.93 (1H, dd, J = 16.8 and 7.7 Hz, C$H_A$$H_B$-3''), 2.99 (1H, dd, J = 16.8 and 4.8 Hz, C$H_A$$H_B$-3''), 3.10-3.31 (3H, m, CH_2-1' and C$H_A$$H_B$-5), 3.70 (3H, s, CH_3O), 3.74 (3H, s, CH_3O), 3.72-3.79 (1H, m, C$H_A$$H_B$-5), 4.18 (1H, m, CH-2), 4.86 (1H, m, CH- 2''). ^{13}C NMR (100 MHz, CH_3OD) δ_C: 14.4 (CH_3, C-11'), 23.7 (CH_2, C-10'), 24.0 (CH_2, C-4), 26.9 (CH_2, C- 2'), 27.6 (CH_2), 30.2 (CH_2), 30.5 (CH_2), 30.5 (CH_2), 30.6 (CH_2), 30.6 (CH_2), 30.7 (CH_2), 33.1 (CH_2, C-9'), 36.4 (CH_2, C-3''), 50.7 (CH, C-2''), 52.6 (CH_3O), 53.2 (CH_3O), 56.3 (CH_2, NCH_2), 56.6 (CH_2, NCH_2), 68.5 (CH, C-2), 169.34 (C=O, NHCO), 171.84 (C=O), 172.13 (C=O). LRMS (ESI), *m/z*: 413 $[M + H]^+$; HRMS (ESI/FT-MS/+ve) m/z: $[M + H]^+$ Calcd for $C_{22}H_{40}N_2O_5$ 413.3010; Found 413.3010.

Dimethyl dodecyl-L-prolyl-L-aspartate dmPLA12

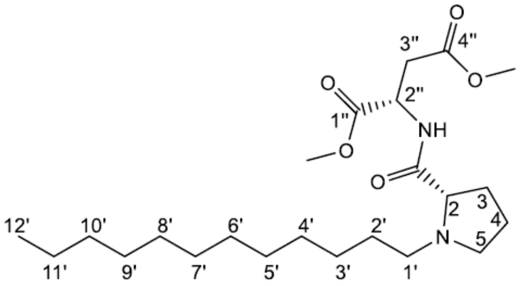

Dimethyl dodecyl-L-prolyl-L-aspartate was synthesised from dodecyl-L-proline (0.85 g, 5.3 mmol) and L-aspartate dimethyl ester (1.5 g, 5.3 mmol) with isobutyl chloroformate (0.8 g, 5.8 mmol) and DIPEA (0.75 g, 5.8 mmol) using the method described above. The crude product was purified by flash column chromatography on silica eluting with ethyl acetate: methanol (80:20 to 0:100), and the final product was obtained as a brown oil (1.24 g, 55%); FTIR (neat, v/cm-1): 3205 (NH, amide), 1735 (C=O, ester), 1681 (C=O, amide), 1172 (C-O, ester), 1002 (C-O, ester); ^{1}H NMR (400 MHz, CD_3OD) δ_H 0.90 (3H, t, J = 6.86 Hz, CH_3-12'), 1. 29-1.36 (18H, m, CH_2-3', CH_2-4', CH_2-5', CH_2-6', CH_2-7', CH_2-8', CH_2-9', CH_2-10', CH_2-11'), 1.67 (2H, m, CH_2-2'), 1.96-2.25 (3H, m, CH_2-4, CH_AH$_B$-3), 2.57 (1H, m, CH$_A$$H_B$-3), 2.92 (1H, dd, J = 16.8 and 7.4 Hz, CH_AH$_B$-3"), 2.99 (1H, dd, J = 16.8 and 4.9 Hz, CH$_A$$H_B$-3"), 3.11-3.27 (3H, m, CH_2-1' and CH_2-5), 3.70 (3H, s, CH_3O), 3.74 (3H, s, CH_3O), 3.71-3.80 (1H, m, CH_2-5), 4.18 (1H, m, CH-2), 4.86 (1H, m, CH-2"); ^{13}C NMR (100 MHz, CH_3OD) δ_C 14.4 (CH_3, C-12'), 23.7 (CH_2, C-11'), 24.0 (CH_2, C-4), 26.8 (CH_2, C-2'), 27.5 (CH_2), 30.1 (CH_2), 30.5 (CH_2), 30.6 (CH_2), 30.7 (CH_2), 30.7 (CH_2), 30.7 (CH_2), 33.1 (CH_2, C-10'), 36.4 (CH_2, C-3"), 50.7 (CH, C-2"), 52.6 (CH_3O), 53.2 (CH_3O), 56.4 (CH_2, NCH_2), 56.6 (CH_2, NCH_2), 68.5 (CH, C-2), 169.2 (C=O, NH*C*O), 171.9 (C=O), 172.1 (C=O); LRMS (ESI), *m/z*: 427 $[M + H]^+$; HRMS (ESI/FT-MS/+ve) m/z: $[M + H]^+$ Calcd for $C_{23}H_{42}N_2O_5$ 427.3167; Found 427.3167.

Dimethyl nonyl-L-prolyl-D-aspartate dmPDA9

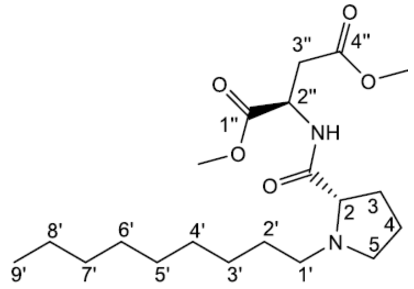

Dimethyl nonyl-L-prolyl-D-aspartate was synthesised from nonyl-L-proline (1 g, 4.2 mmol) and D-aspartate dimethyl ester (0.67 g, 4.2 mmol) with isobutyl chloroformate (0.63 g, 4.6 mmol) and DIPEA

(0.6 g, 4.6 mmol) using the method described above. The crude product was purified by flash column chromatography on silica eluting with ethyl acetate: methanol (75:25 to 0:100), and the final product was obtained as a brown oil (0.94 g, 59%); FTIR (neat, v/cm-1): 3178 (NH, amide), 1743 (C=O, ester), 1681 (C=O, amide), 1172 (C-O, ester), 1002 (C-O, ester); ^{1}H NMR (400 MHz, CD_3OD) δ_H 0.90 (3H, t, J = 6.40 Hz, CH_3-9'), 1.27-1.31 (12H, m, CH_2-3', CH_2-4', CH_2-5', CH_2-6', CH_2-7', CH_2-8'), 1.68 (2H, m, CH_2-2'), 2.03 (2H, m, CH_2-4), 2.18 (1H, m, C$H_A$$H_B$-3), 2.51 (1H, m, C$H_A$$H_B$-3), 2.92 (1H, dd, J = 16.8 and 7.6 Hz, C$H_A$$H_B$-3''), 2.98 (1H, dd, J = 16.8 and 4.8 Hz, C$H_A$$H_B$-3''), 3.13-3.27 (3H, m, CH_2-1' and CH_2-5), 3.70 (3H, s, CH_3O), 3.75 (3H, s, CH_3O), 3.70-3.79 (1H, m, CH_2-5), 4.16 (1H, m, CH-2), 4.85 (1H, m, CH-2''); ^{13}C NMR (100 MHz, CH_3OD) δ_C 14.4 (CH_3, C-9'), 23.7 (CH_2, C-8'), 24.0 (CH_2, C-4), 26.8 (CH_2, C-2'), 27.6 (CH_2), 30.1 (CH_2), 30.3 (CH_2), 30.4 (CH_2), 30.7 (CH_2), 33.0 (CH_2, C-8'), 36.4 (CH_2, C-3''), 50.6 (CH, C-2''), 52.6 (CH_3O), 53.3 (CH_3O), 56.3 (CH_2, NCH_2), 56.7 (CH_2, NCH_2), 68.6 (CH, C-2), 169.3 (C=O, NH*C*O), 171.9 (C=O), 172.1 (C=O); LRMS (ESI), *m/z*: 385 $[M + H]^+$; HRMS (ESI/FT-MS/+ve) m/z: $[M + H]^+$ Calcd for $C_{20}H_{36}N_2O_5$ 385.2697; Found 385.2697.

Dimethyl decyl-L-prolyl-D-aspartate dmPDA10

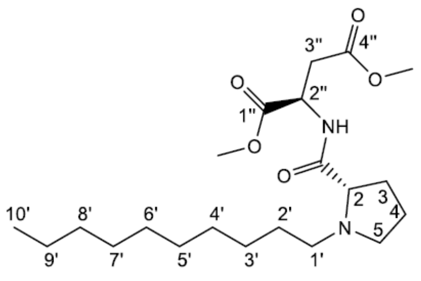

Dimethyl decyl-L-prolyl-D-aspartate was synthesised from decyl-L-proline (1.07 g, 4.2 mmol) and D-aspartate dimethyl ester (0.68 g, 4.2 mmol) with isobutyl chloroformate (0.63 g, 4.6 mmol) and DIPEA (0.6 g, 4.6 mmol) using the method described above. The crude product was purified by flash column chromatography on silica eluting with ethyl acetate: methanol (75:25 to 0:100), and the final product was obtained as a brown oil (1.12 g, 67%); FTIR (neat, v/cm-1): 3186 (NH, amide), 1739 (C=O, ester), 1681 (C=O, amide), 1170 (C-O, ester), 1001 (C-O, ester); ^{1}H NMR (400 MHz, CD_3OD) δ_H 0.90 (3H, t, J = 6.84 Hz, CH_3-10'), 1.31-1.36 (14H, m, CH_2-3', CH_2-4', CH_2-5', CH_2-6', CH_2-7', CH_2-8', CH_2-9'), 1.68 (2H, m, CH_2-2'), 2.03 (2H, m, CH_2-4), 2.18 (1H, m, C$H_A$$H_B$-3), 2.51 (1H, m, C$H_A$$H_B$-3), 2.90 (1H, dd, J = 16.8 and 7.4 Hz, C$H_A$$H_B$-3''), 2.98 (1H, dd, J = 16.8 and 4.9 Hz, C$H_A$$H_B$-3''), 3.13-3.25 (3H, m, CH_2-1' and CH_2-5), 3.70 (3H, s, CH_3O), 3.75 (3H, s, CH_3O), 3.74-3.80 (1H, m, CH_2-5), 4.17 (1H, m, CH-2), 4.83 (1H, m, CH- 2''); ^{13}C NMR (100 MHz, CH_3OD) δ_C 14.4 (CH_3, C-10'), 23.7 (CH_2, C-9'), 24.0 (CH_2, C-4), 26.9 (CH_2, C-2'), 27.6 (CH_2), 30.1 (CH_2), 30.4 (CH_2), 30.5 (CH_2), 30.6 (CH_2), 33.0 (CH_2, C-8'), 36.4 (CH_2, C-3''), 50.6 (CH, C- 2''), 52.6 (CH_3O), 53.3 (CH_3O), 56.3 (CH_2, NCH_2), 56.6 (CH_2, NCH_2), 68.5

(CH, C-2), 169.5 (C=O, NH*C*O), 171.8 (C=O), 172.1 (C=O); LRMS (ESI), *m/z*: 399 $[M + H]^+$; HRMS (ESI/FT-MS/+ve) m/z: $[M + H]^+$ Calcd for $C_{21}H_{38}N_2O_5$ 399.2854; Found 399.2854.

Dimethyl undecyl-L-prolyl-D-aspartate dmPDA11

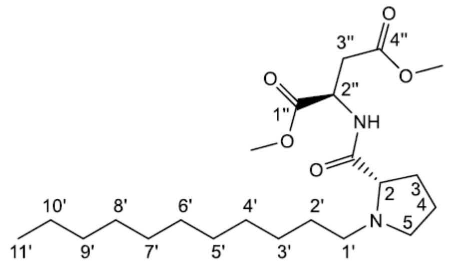

Dimethyl undecyl-L-prolyl-D-aspartate was synthesised from undecyl-L-proline (1 g, 3.7 mmol) and D-aspartate dimethyl ester (0.6 g, 3.7 mmol) with isobutyl chloroformate (0.56 g, 4.1 mmol) and DIPEA (0.53 g, 4.1 mmol) using the method described above. The crude product was purified by flash column chromatography on silica eluting with ethyl acetate: methanol (75:25 to 0:100), and the final product was obtained as a brown oil (0.8 g, 52%); FTIR (neat, v/cm-1): 3215 (NH, amide), 1739 (C=O, ester), 1683 (C=O, amide), 1170 (C-O, ester), 999 (C-O, ester); ^1H NMR (400 MHz, CD_3OD) δ_H 0.90 (3H, t, J = 6.9 Hz, CH_3-11'), 1.22-1.44 (16H, m, CH_2-3', CH_2-4', CH_2-5', CH_2-6', CH_2-7', CH_2-8', CH_2-9', CH_2-10'), 1.68 (2H, m, CH_2-2'), 1.97-2.10 (2H, m, CH_2-4), 2.10-2.24 (1H, m, CH_AH_B-3), 2.45-2.61 (1H, m, CH_AH_B-3), 2.90 (1H, dd, J = 16.8 and 7.4 Hz, CH_AH_B-3"), 2.97 (1H, dd, J = 16.8 and 4.9 Hz, CH_AH_B-3"), 3.14-3.26 (3H, m, CH_2-1' and CH_AH_B-5) , 3.70 (3H, s, CH_3O), 3.75 (3H, s, CH_3O), 3.70-3.79 (1H, m, CH_AH_B-5), 4.18 (1H, m, CH-2), 4.83 (1H, m, CH-2"); ^{13}C NMR (100 MHz, CH_3OD) δ_C 14.4 (CH_3, C-11'), 23.7 (CH_2, C-10'), 24.0 (CH_2, C-4), 26.8 (CH_2, C-2'), 27.6 (CH_2), 30.1 (CH_2), 30.5 (CH_2), 30.5 (CH_2), 30.6 (CH_2), 30.6 (CH_2), 33.1 (CH_2, C- 9'), 36.4 (CH_2, C-3"), 50.6 (CH, C-2"), 52.6 (CH_3O), 53.3 (CH_3O), 56.3 (CH_2, NCH_2), 56.6 (CH_2, NCH_2), 68.6 (CH, C-2), 169.3 (C=O, NH*C*O), 171.8 (C=O), 172.1 (C=O); LRMS (ESI), *m/z*: 413 $[M + H]^+$; HRMS (ESI/FT-MS/+ve) m/z: $[M + H]^+$ Calcd for $C_{22}H_{40}N_2O_5$ 413.3010; Found 413.3010.

Dimethyl dodecyl-L-prolyl-D-aspartate dmPDA12

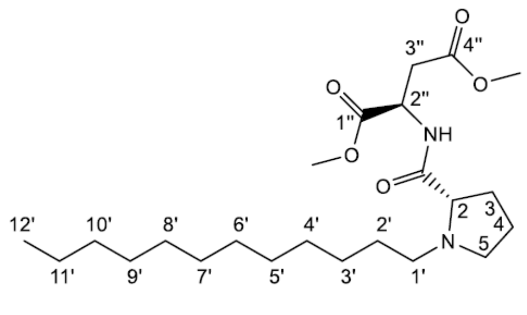

Dimethyl dodecyl-L-prolyl-D-aspartate was synthesised from dodecyl-L-proline (1.5 g, 5.3 mmol) and D-aspartate dimethyl ester (0.85 g, 5.3 mmol) with isobutyl chloroformate (0.8 g, 5.58 mmol) and DIPEA (0.75 g, 5.8 mmol) using the method described above. The crude product was purified by flash column chromatography on silica eluting with ethyl acetate: methanol (80:20 to 0:100), and the final product was obtained as a brown oil (1.08 g, 47%); FTIR (neat, v/cm-1): 3367 (NH, amide), 1740 (C=O, ester), 1678 (C=O, amide), 1172 (C-O, ester), 976 (C-O, ester); ^{1}H NMR (400 MHz, CD_3OD) δ_H 0.90 (3H, t, J = 6.8 Hz, CH_3-12'), 1.22-1.43 (18H, m, CH_2-3', CH_2-4', CH_2-5', CH_2-6', CH_2-7', CH_2-8', CH_2-9', CH_2-10', CH_2-10'), 1.68 (2H, m, CH_2-2'), 2.03 (2H, m, CH_2-4), 2.18 (1H, m, CH_AH$_B$-3), 2.53 (1H, m, CH$_A$$H_B$-3), 2.92 (1H, dd, J = 16.8 and 7.4 Hz, CH_AH$_B$-3''), 2.98 (1H, dd, J = 16.8 and 4.9 Hz, CH$_A$$H_B$-3''), 3.10-3.27 (3H, m, CH_2-1' and CH_AH$_B$-5) , 3.70 (3H, s, CH_3O), 3.75 (3H, s, CH_3O), 3.70-3.80 (1H, m, CH$_A$$H_B$-5), 4.17 (1H, m, CH-2), 4.87 (1H, m, CH-2''); ^{13}C NMR (100 MHz, CH_3OD) δ_C 14.4 (CH_3, C-12'), 23.7 (CH_2, C-11'), 24.0 (CH_2, C-4), 26.8 (CH_2, C-2'), 27.6 (CH_2), 30.1 (CH_2), 30.4 (CH_2), 30.5 (CH_2), 30.6 (CH_2), 30.7 (CH_2), 30.7 (CH_2), 33.1 (CH_2, C-9'), 36.4 (CH_2, C-3''), 50.7 (CH, C-2''), 52.6 (CH_3O), 53.3 (CH_3O), 56.3 (CH_2, NCH_2), 56.7 (CH_2, NCH_2), 68.6 (CH, C-2), 169.3 (C=O, NH*C*O), 171.8 (C=O), 172.1 (C=O); LRMS (ESI), *m/z*: 427 $[M + H]^+$; HRMS (ESI/FT-MS/+ve) m/z: $[M + H]^+$ Calcd for $C_{23}H_{42}N_2O_5$ 427.3167; Found 427.3167.

General procedure for the hydrolysis to give PLA9 & 11 and PDA9, 11 & 12

Ester (1 equiv.) was dissolved in methanol (5 mL) and left to stir at room temperature. LiOH (4 equiv.) was added to the reaction mixture followed by 1 mL of water. The reaction was refluxed for 1 hour. Upon completion of reaction, solvent was removed *in vacuo*, and the residue was taken up in water. The pH was adjusted to 2 with 1M HCl and extracted with ethyl acetate. The combined organic layers were dried over anhydrous Na_2SO_4 and reduced *in vacuo* to give final product.

Nonyl-L-prolyl-L-aspartate PLA9

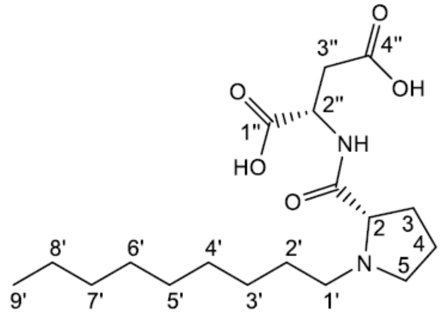

Nonyl-L-prolyl-L-aspartate was synthesised from dimethyl nonyl-L-prolyl-L-aspartate (0.3 g, 0.08 mmol) and LiOH (0.075g, 3.1 mmol) using the method above. The product was obtained as a brown oil (98 mg, 35%); FTIR (neat, v/cm-1): 3209 (NH, amide), 1724 (C=O, carboxylic acid), 1670 (C=O, amide), 1215 (C-O, carboxylic acid); ^{1}H NMR (400 MHz, CD3OD) δ_H 0.90 (3H, t, J = 6.8 Hz, CH_3-9'), 1.24-1.43 (12H, m, CH_2-3', CH_2-4', CH_2-5', CH_2-6', CH_2-7', CH_2-8'), 1.68 (2H, m, CH_2-2'), 1.98-2.24 (3H, m, CH_2-4, C$H_A$$H_B$-3), 2.55 (1H, m, $CH_A$$H_B$-3), 2.87 (1H, dd, J = 16.8 and 7.6 Hz, C$H_A$$H_B$-3''), 2.95 (1H, dd, J = 16.8 and 4.7 Hz, $CH_A$$H_B$-3''), 3.12-3.26 (3H, m, CH_2-1' and C$H_A$$H_B$-5), 3.74 (1H, m, $CH_A$$H_B$-5), 4.16 (1H, m, CH-2), 4.76 (1H, m, CH-2''); ^{13}C NMR (100 MHz, CH_3OD) δ_C 14.4 (CH_3, C-9'), 23.7 (CH_2, C-8'), 24.0 (CH_2, C-4), 26.9 (CH_2, C-2'), 27.6 (CH_2), 30.1 (CH_2), 30.3 (CH_2), 30.5 (CH_2), 30.5 (CH_2), 33.0 (CH_2), 36.7 (CH_2, C-3''), 50.9 (CH, C- 2''), 56.2 (CH_2, NCH_2), 56.5 (CH_2, NCH_2), 68.6 (CH, C-2), 169.1 (C=O, NH*C*O), 173.5 (C=O), 173.8 (C=O). LRMS (ESI), *m/z*: 357 $[M + H]^+$; HRMS (ESI/FT-MS/+ve) m/z: $[M + H]^+$ Calcd for $C_{18}H_{32}N_2O_5$ 357.2384; Found 357.2384.

Undecyl-L-prolyl-L-aspartate PLA11

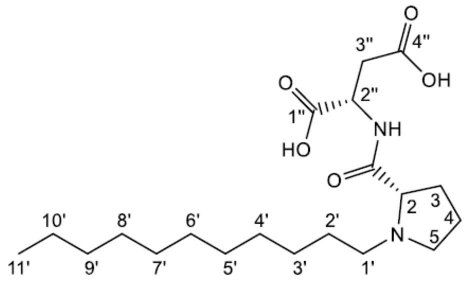

Undecyl-L-prolyl-L-aspartate was synthesised from dimethyl undecyl-L-prolyl-L-aspartate (0.2 g, 0.05 mmol) and LiOH (0.046 g, 0.2 mmol) using the method above. The product was obtained as a brown oil (0.12 g, 62%); FTIR (neat, v/cm-1): 3240 (NH, amide), 1728 (C=O, carboxylic acid), 1674 (C=O, amide), 1219 (C-O, carboxylic acid); ^{1}H NMR (400 MHz, CD_3OD) δ_H 0.90 (3H, t, J = 6.8 Hz, CH_3-11'), 1.23-1.43 (16H, m, CH_2-3', CH_2-4', CH_2-5', CH_2-6', CH_2-7', CH_2-8', CH_2-9', CH_2-10'), 1.68 (2H, m, CH_2-2'), 1.98-2.24 (3H, m, CH_2-4, C$H_A$$H_B$-3), 2.54 (1H, m, $CH_A$$H_B$-3), 2.90 (1H, dd, J = 16.8 and 7.4 Hz, C$H_A$$H_B$-

3''), 2.97 (1H, dd, J = 16.8 and 4.9 Hz, CH_AH_B-3''), 3.07-3.27 (3H, m, CH_2-1' and CH_AH_B-5), 3.75 (1H, m, CH_AH_B-5), 4.17 (1H, m, CH-2), 4.77 (1H, m, CH-2''); ^{13}C NMR (100 MHz, CH_3OD) δ_C 14.4 (CH_3, C-11'), 23.7 (CH_2, C-10'), 24.0 (CH_2, C-4), 26.8 (CH_2, C-2'), 27.6 (CH_2), 27.6 (CH_2), 30.1 (CH_2), 30.5 (CH_2), 30.5 (CH_2), 30.6 (CH_2), 30.7 (CH_2), 33.1 (CH_2), 36.6 (CH_2, C-3''), 50.8 (CH, C-2''), 56.2 (CH_2, NCH_2), 56.5 (CH_2, NCH_2), 68.6 (CH, C-2), 169.1 (C=O, NH*C*O), 173.4 (C=O), 173.7 (C=O); LRMS (ESI), *m/z*: 385 $[M + H]^+$; HRMS (ESI/FT-MS/+ve) m/z: $[M + H]^+$ Calcd for $C_{20}H_{36}N_2O_5$ 385.2697; Found 385.2697.

Dodecyl-L-prolyl-L-aspartate PDA12

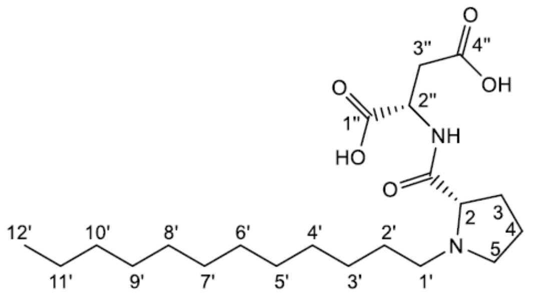

Dodecyl-L-prolyl-L-aspartate was synthesised from dimethyl dodecyl-L-prolyl-L-aspartate (0.2g, 0.05 mmol) and LiOH (0.045g, 0.2 mmol) using the method above. The product was obtained as a brown oil (0.088 g, 44%); FTIR (neat, v/cm-1): 3255 (NH, amide), 1728 (C=O, carboxylic acid), 1674 (C=O, amide), 1219 (C-O, carboxylic acid); ^1H NMR (400 MHz, CD_3OD) δ_H 0.90 (3H, t, J = 6.8 Hz, CH_3-12'), 1.23- 1.43 (18H, m, CH_2-3', CH_2-4', CH_2-5', CH_2-6', CH_2-7', CH_2-8', CH_2-9', CH_2-10', CH_2-11'), 1.68 (2H, m, CH_2- 2'), 1.98-2.23 (3H, m, CH_2-4, CH_AH_B-3), 2.55 (1H, m, CH_AH_B-3), 2.90 (1H, dd, J = 16.8 and 7.4 Hz, CH_AH_B- 3''), 2.97 (1H, dd, J = 16.8 and 4.9 Hz, CH_AH_B-3''), 3.09-3.287 (3H, m, CH_2-1' and CH_AH_B-5), 3.75 (1H, m, CH_AH_B-5), 4.17 (1H, m, CH-2), 4.80 (1H, m, CH-2''); ^{13}C NMR (100 MHz, CH_3OD) δ_C 14.4 (CH_3, C-12'), 23.7 (CH_2, C-11'), 24.0 (CH_2, C-4), 26.8 (CH_2, C-2'), 27.6 (CH_2), 27.6 (CH_2), 30.1 (CH_2), 30.5 (CH_2), 30.5 (CH_2), 30.5 (CH_2), 30.6 (CH_2), 30.7 (CH_2), 33.1 (CH_2), 36.6 (CH_2, C-3''), 50.8 (CH, C-2''), 56.2 (CH_2, NCH_2), 56.2 (CH_2, NCH_2), 68.6 (CH, C-2), 169.1 (C=O, NH*C*O), 173.3 (C=O), 173.7 (C=O). LRMS (ESI), *m/z*: 400 $[M + H]^+$; HRMS (ESI/FT-MS/+ve) m/z: $[M + H]^+$ Calcd for $C_{21}H_{38}N_2O_5$ 399.2854; Found 399.2854.

Nonyl-L-prolyl-D-aspartate PDA9

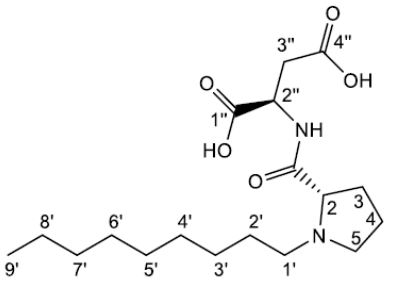

Nonyl-L-prolyl-D-aspartate was synthesised from dimethyl nonyl-L-prolyl-D-aspartate (0.3 g, 0.08 mmol) and LiOH (0.075g, 0.3 mmol) using the method above. The product was obtained as a brown oil (0.14 g, 51%); FTIR (neat, v/cm-1): 3217 (NH, amide), 1724 (C=O, carboxylic acid), 1674 (C=O, amide), 1215 (C-O, carboxylic acid); ^{1}H NMR (400 MHz, CD_3OD) δ_H 0.90 (3H, t, J = 7.0 Hz, CH_3-9'), 1.24-1.43 (14H, m, CH_2-3', CH_2-4', CH_2-5', CH_2-6', CH_2-7', CH_2-8',), 1.70 (2H, m, CH_2-2'), 2.04 (2H, m, CH_2-4), 2.17 (1H, m, CH_AH_B-3), 2.51 (1H, m, CH_AH_B-3), 2.85 (1H, dd, J = 16.8 and 7.8 Hz, CH_AH_B-3"), 2.95 (1H, dd, J = 16.8 and 4.7 Hz, CH_AH_B-3"), 3.11-3.28 (3H, m, CH_2-1' and CH_AH_B-5), 3.75 (1H, m, CH_AH_B-5), 4.17 (1H, m, CH-2), 4.77 (1H, m, CH-2"); ^{13}C NMR (100 MHz, CH_3OD) δ_C 14.4 (CH_3, C-9'), 23.7 (CH_2, C-8'), 24.0 (CH_2, C-4), 26.8 (CH_2, C-2'), 27.6 (CH_2), 30.1 (CH_2), 30.3 (CH_2), 30.5 (CH_2), 30.7 (CH_2), 33.0 (CH_2), 36.9 (CH_2, C- 3"), 50.9 (CH, C-2"), 56.2 (CH_2, NCH_2), 56.6 (CH_2, NCH_2), 68.7 (CH, C-2), 169.2 (C=O, NH*C*O), 173.6 (C=O), 173.8 (C=O). LRMS (ESI), *m/z*: 357 $[M + H]^+$; HRMS (ESI/FT-MS/+ve) m/z: $[M + H]^+$ Calcd for $C_{18}H_{32}N_2O_5$ 357.2384; Found 357.2384.

Undecyl-L-prolyl-D-aspartate PDA11

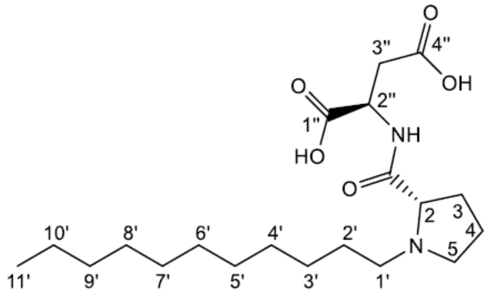

Undecyl-L-prolyl-D-aspartate was synthesised from dimethyl undecyl-L-prolyl-D-aspartate (0.4 g, 0.1 mmol) and LiOH (0.092 g, 0.4 mmol) using the method above. The product was obtained as a brown oil (0.15 g, 39%); FTIR (neat, v/cm-1): 3360 (NH, amide), 1720 (C=O, carboxylic acid), 1674 (C=O, amide), 1226 (C-O, carboxylic acid); ^{1}H NMR (400 MHz, CD_3OD) δ_H 0.90 (3H, t, J = 6.8 Hz, CH_3-11'), 1.23-1.44 (16H, m, CH_2-3', CH_2-4', CH_2-5', CH_2-6', CH_2-7', CH_2-8', CH_2-9', CH_2-10'), 1.70 (2H, m, CH_2-2'), 2.04 (2H, m, CH_2-4), 2.18 (1H, m, CH_AH_B-3), 2.53 (1H, m, CH_AH_B-3), 2.87 (1H, dd, J = 16.8 and 7.8 Hz, CH_AH_B-3"), 2.95 (1H, dd, J = 16.8 and 4.6 Hz, CH_AH_B-3"), 3.12-3.27 (3H, m, CH_2-1' and CH_AH_B-5), 3.75 (1H, m, CH_AH_B- 5), 4.22 (1H, m, CH-2), 4.80 (1H, m, CH-2"); ^{13}C NMR (100 MHz, CH_3OD) δ_C

14.5 (CH_3, C-11'), 23.7 (CH_2, C-10'), 24.0 (CH_2, C-4), 26.8 (CH_2, C-2'), 27.6 (CH_2), 30.1 (CH_2), 30.2 (CH_2), 30.4 (CH_2), 30.5 (CH_2), 30.6 (CH_2), 30.7 (CH_2), 33.0 (CH_2), 36.6 (CH_2, C-3''), 50.7 (CH, C-2''), 56.3 (CH_2, NCH_2), 56.6 (CH_2, NCH_2), 68.7 (CH, C-2), 169.2 (C=O, NH*C*O), 173.2 (C=O), 173.6 (C=O); LRMS (ESI), *m/z*: 385 $[M + H]^+$; HRMS (ESI/FT-MS/+ve) m/z: $[M + H]^+$ Calcd for $C_{20}H_{36}N_2O_5$ 385.2697; Found 385.2697.

Dodecyl-L-prolyl-D-aspartate PDA12

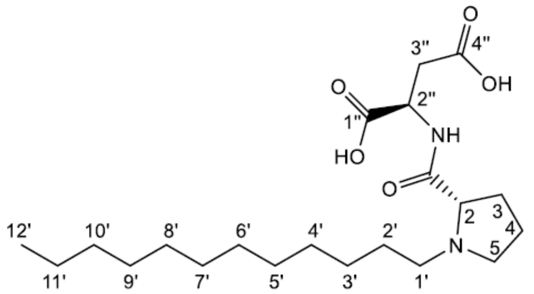

Dodecyl-L-prolyl-D-aspartate was synthesised from dimethyl dodecyl-L-prolyl-D-aspartate (0.2 g, 0.05 mmol) and LiOH (0.045g, 0.2 mmol) using the method above. The product was obtained as a yellow oil (0.12 g, 66%); FTIR (neat, v/cm-1): 3205 (NH, amide), 1728 (C=O, carboxylic acid), 1678 (C=O, amide), 1215 (C-O, carboxylic acid); ^1H NMR (400 MHz, CD_3OD) δ_H 0.90 (3H, t, J = 6.8 Hz, CH_3-12'), 1.24-1.44 (18H, m, CH_2-3', CH_2-4', CH_2-5', CH_2-6', CH_2-7', CH_2-8', CH_2-9', CH_2-10', CH_2-11'), 1.70 (2H, m, CH_2-2'), 2.04 (2H, m, CH_2-4), 2.16 (1H, m, CH_AH_B-3), 2.52 (1H, m, CH_AH_B-3), 2.87 (1H, dd, J = 16.8 and 7.8 Hz, CH_AH_B-3''), 2.97 (1H, dd, J = 16.8 and 4.6 Hz, CH_AH_B-3''), 3.08-3.28 (3H, m, CH2-1' and CH_AH_B-5), 3.77 (1H, m, CH_AH_B-5), 4.17 (1H, m, CH-2), 4.80 (1H, m, CH-2''); ^{13}C NMR (100 MHz, CH_3OD) δ_C 14.4 (CH_3, C- 12'), 23.7 (CH_2, C-11'), 24.0 (CH_2, C-4), 26.8 (CH_2, C-2'), 27.6 (CH_2), 29.9 (CH_2), 30.1 (CH_2), 30.2 (CH_2), 30.5 (CH_2), 30.5 (CH_2), 30.6 (CH_2), 30.7 (CH_2), 33.1 (CH_2), 36.6 (CH_2, C-3''), 50.6 (CH, C-2''), 56.2 (CH_2, NCH_2), 56.6 (CH_2, NCH_2), 68.7 (CH, C-2), 169.1 (C=O, NH*C*O), 173.2 (C=O), 173.6 (C=O). LRMS (ESI), *m/z*: 399 $[M + H]^+$; HRMS (ESI/FT-MS/+ve) m/z: $[M + H]^+$ Calcd for $C_{21}H_{38}N_2O_5$ 399.2854; Found 399.2854.

Synthesis of 1-Decyl-3-(2-hydroxy-2,2-diphosphonoethyl)-1H-imidazol-3-ium (10-ZOL)

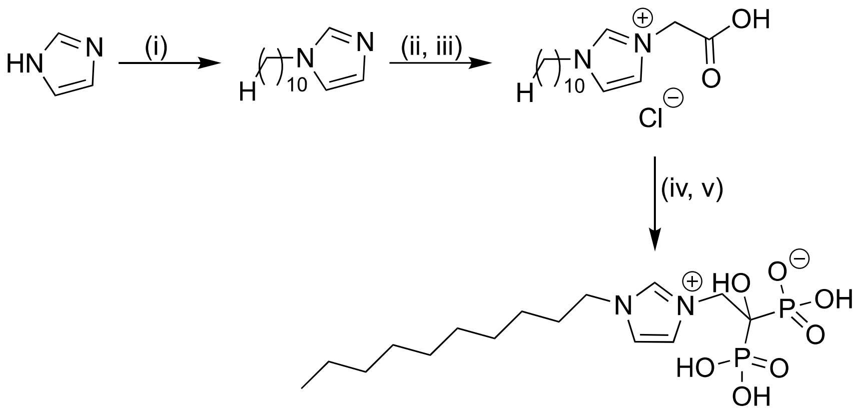

10-ZOL

Scheme 2

Reagents and conditions: (i) 1-bromodecane (1equiv.), K_2CO_3 (2 equiv.), acetone, reflux, 48 h; (ii) methyl bromoacetate (1 equiv.), EtOAc, rt, 24 h; (iii) HCl (6M), reflux, 24 h; (iv) PCl_3 (3 equiv.), toluene, 80 °C, 24 h; (v) HCl (6M), reflux, 24 h.

Imidazole was alkylated with 1-bromodecane to give 1-decylimidazole. The success of the reaction was confirmed by the appearance of 3 singlets, at δ6.89, δ7.05 and δ7.45, in the ^{1}H NMR spectrum, as well as the three peaks in the ^{13}C spectrum (at δ118.9, δ129.5 and δ137.2), which correspond to the three protons and carbons of the imidazole ring, as well as signals for the aliphatic chain. 1-Decylimidazole was then alkylated using methyl bromoacetate to give an imidazolium salt, which was then hydrolysed under acidic conditions using aq. HCl (6M) to give carboxylic acid. The ^{1}H and ^{13}C NMR spectra of acid show the appearance of new peaks at δ5.13 and δ51.7, respectively, that correspond to the additional methylene group, confirming that the alkylation and sequential hydrolysis were successful. Using a method adapted from Zhang et al. the carboxylic acid was first heated to 80 °C in toluene, then reacted with PCl_3 (3 equiv.) overnight, followed by hydrolysis with aq. HCl (6M) overnight. The synthesis of bisphosphonic acid was confirmed by analysis of the ^{1}H NMR spectrum 10-ZOL; the 1H NMR peaks for the methylene being transformed from a singlet to a triplet due to the coupling to the two phosphorus nuclei. The ^{31}P NMR spectrum also displays a peak at δ13.93, indicating the presence of the two phosphonate groups.

1-Decyl-1H-imidazole

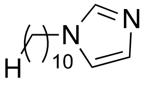

A mixture of imidazole (3.4 g, 0.05 mol, 1 equiv.), 1-bromodecane (11.1 g, 0.05 mol, 1 equiv.) and K_2CO_3 (13.8 g, 0.1 mol, 2 equiv.) in acetone (200 mL) was refluxed overnight. Upon filtration and removal of solvent in vacuo, the crude product was purified via flash chromatography, eluting with ethyl acetate: hexane (20:80 to 100:0) to give the desired product as a yellow oil (9.1 g, 97%); ^{1}H NMR (400 MHz, $CDCl_3$) δ_H: 0.87 (3H, t, J = 6.9 Hz, CH3-10), 1.25 (14H, m, CH2-3’, CH2-4’, CH2-5’, CH2-6’, CH2-7’, CH2-8’, CH2-9’), 1.76 (2H, m, CH2-2’), 3.91 (2H, t, J = 7.2 Hz, CH2-1’), 6.89 (1H, s, CH-2), 7.05 (1H, s, CH-3), 7.45 (1H, s, CH-1); ^{13}C NMR (100 MHz, CDCl3) δ_C 14.2 (CH_3, C-10’), 22.8 (CH_2), 26.7 (CH_2), 29.2 (CH_2), 29.4 (CH_2), 39.6 (CH_2), 29.6 (CH_2), 31.2 (CH_2, C-2’), 32.0 (CH_2), 47.2 (CH_2, C-1’), 118.9 (CH, C-2), 129.5 (CH, C-3), 137.2 (CH, C-1); LRMS (ESI), m/z: 209 $[M + H]^+$.

3-(Carboxymethyl)-1-decyl-1*H*-imidazol-3-ium Chloride

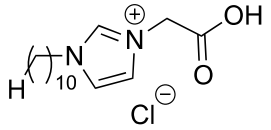

1-Decyl-1H-imidazole (5.2 g, 0.025 mol, 1 equiv.) was stirred with methyl bromoacetate (3.8 g, 0.025 mol, 1 equiv.) in ethyl acetate (100 mL) at room temperature overnight to give imidazolium salt. After 24 hours, solvent was removed in vacuo and the residue was refluxed in 6M HCl (10 mL) to give the crude product which was reduced under pressure and purified by recrystallisation in water/acetone. The carboxylic acid was obtained as a white solid (6.1 g, 100 %). ^{1}H NMR (400 MHz, D_2O) δ_H 0.90 (3H, t, J = 6.9 Hz, CH3-10), 1.30-1.37 (14H, m, CH2-3’, CH2-4’, CH2-5’, CH2-6’, CH2-7’, CH2-8’, CH2-9’), 1.91 (2H, m, CH2-2’), 4.21 (2H, t, J = 7.3 Hz, CH2-1’), 5.13 (2H, s, CH2-1”), 7.64 (1H, s, NCH), 7.70 (1H, s, NCH), 9.05 (1H, s, CH-1); ^{13}C NMR (100 MHz, D_2O) δC 14.4 (CH_3, C-10’), 23.7 (CH_2), 27.2 (CH_2), 30.1 (CH_2), 30.4 (CH_2), 30.5 (CH_2), 30.6 (CH_2), 31.1 (CH_2, C-2’), 33.0 (CH_2), 50.8 (CH_2, NCH2), 51.7 (CH_2, NCH2), 123.3 (CH, NCH), 125.2 (CH, NCH), 138.6 (CH, C-1), 169.1 (C-2”); LRMS (ESI), m/z: 267 M^+.

1-Decyl-3-(2-hydroxy-2,2-diphosphonoethyl)-1H-imidazol-3-ium (10-ZOL)

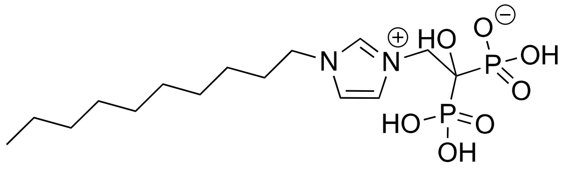

3-(Carboxymethyl)-1-decyl-1*H*-imidazol-3-ium (1.73g, 6 mmol) in toluene (15 ml) was heat to 80°C with stirring. After all solids had melted PCl_3 (2.4g, 18 mmol) was added dropwise over 10 minutes and heating was continued at 80°C for 12 h. After cooling to room temperature, the toluene was decanted from the residue which was refluxed in aqueous HCl (6M, 20 mL) for 24 h. After cooling to room temperature, most of the solvent was removed under reduced pressure and the residue dissolved in a minimum amount of water and acetone added until the solution became cloudy and was cooled to 4°C to precipitate the 3-(carboxymethyl)-1-dodecyl-1H-imidazol-3-ium, which was isolated by filtration, washed with 2-propanol (5 x 5 mL), dried, and further purified by recrystallization from H_2O/*i*-PrOH to afford pure 1-hydroxy-2-(N-decyl-imidazolium-1-yl)ethylidene-1,1-bisphosphonic acid hydrochloride as a white solid (1.8 g, 67 %). ^{1}H NMR (400 MHz, D_2O) δH 0.86 (3H, t, J = 6.8 Hz, CH3-10'), 1.27-1.32 (14H, m, CH2-3', CH2-4', CH2-5', CH2-6', CH2-7', CH2-8', CH2-9'), 1.87 (2H, m, CH2-2'), 4.20 (2H, t, J = 7.2 Hz, CH2-1'), 4.66 (2H, t, JPH = 9.7 Hz, CH2-1''), 7.42 (1H, s, NCH), 7.52 (1H, s, NCH), 8.77 (1H, s, CH-1); ^{13}C NMR (100 MHz, D_2O) δ_C 13.3 (CH_3, C-10'), 22.0 (CH_2), 25.2 (CH_2), 27.9 (CH_2), 28.3 (CH_2), 28.4 (CH_2), 28.5 (CH_2), 29.1 (CH_2, C-2'), 31.1 (CH_2), 49.5 (CH_2, C-1'), 52.6 (CH_2, C-1''), 72.7 (t, J = 133.8 Hz, C-2''), 128.9 (CH, NCH), 124.3 (CH, NCH), 136.5 (C-1); ^{31}P NMR (161 MHz, D_2O) δ: 13.93; LRMS (ESI), m/z: 413 M^+; HRMS (ESI/FT-MS/+ve) m/z: M^+ Calcd for $C_{15}H_{31}N_2O_7P_2$ 413.1601; Found 413.1600.

Zhang, Y.; Cao, R.; Yin, F.; Hudock, M. P.; Guo, R.-T.; Krysiak, K.; Mukherjee, S.; Gao, Y.-G.; Robinson, H.; Song, Y., Lipophilic bisphosphonates as dual farnesyl/geranylgeranyl diphosphate synthase inhibitors: an X-ray and NMR investigation. *Journal of the American Chemical Society* **2009,** *131* (14), 5153-5162.
